# Intracellular Calcium Overload Promotes NFATc1-ATF3 Activation and Induces the Senescence-Associated Phenotype in Irradiated Osteocytes

**DOI:** 10.3390/life16060984

**Published:** 2026-06-11

**Authors:** Haiqing Han, Fanyu Zhao, Jianping Wang, Jianglong Zhai, Guoying Zhu

**Affiliations:** Institute of Radiation Medicine, Fudan University, 2094 Xietu Road, Shanghai 200032, China; 23211140001@m.fudan.edu.cn (H.H.); 22211140009@m.fudan.edu.cn (F.Z.); jianpingwang@fudan.edu.cn (J.W.); jlzhai@fudan.edu.cn (J.Z.)

**Keywords:** calcium overload, osteocyte senescence, ionizing radiation, NFATc1, ATF3, bone homeostasis

## Abstract

Although calcium overload dysregulation has been implicated in cellular senescence, its role in ionizing radiation (IR)-induced osteocyte senescence, a key pathogenic mechanism underlying radiotherapy-associated bone injury, remains poorly explored. This study investigated whether IR-induced osteocyte senescence is mediated through the Ca^2+^-NFATc1-ATF3 pathway. Exposure to 2 Gy X-rays impaired osteocyte homeostasis, manifesting as reduced viability and proliferation, G2/M phase arrest, and dendritic retraction. IR also induced persistent DNA damage response and senescence-associated phenotypes, including increased γ-H2AX foci, SA-β-gal activity, condensed punctate DAPI-dense nuclear foci, p16/p21 expression, and pro-inflammatory SASP profile. Intracellular Ca^2+^ levels surged within 6 h post-irradiation and remained elevated for at least 72 h in a dose-dependent manner. Pharmacological Ca^2+^ modulation with BAPTA-AM or verapamil attenuated IR-induced intracellular Ca^2+^ accumulation, G2/M arrest, SA-β-gal positivity, p21/p53 upregulation, and SASP secretion. Conditioned medium from irradiated osteocytes inhibited BMSC-mediated osteogenesis and enhanced BMM-driven osteoclastogenesis, whereas Ca^2+^ modulation partially mitigated these paracrine effects. Mechanistically, IR promoted NFATc1 nuclear translocation and ATF3 upregulation. Collectively, these findings support an important role for pathological intracellular Ca^2+^ elevation in IR-induced osteocyte senescence and suggest that the Ca^2+^-NFATc1-ATF3 axis may represent a potential therapeutic target for mitigating radiation-associated disruption of bone homeostasis.

## 1. Introduction

Ionizing radiation (IR) is a cornerstone modality in the management of primary and metastatic bone tumors; however, its therapeutic benefits are invariably accompanied by detrimental effects on adjacent normal tissues, including the skeleton [1]. Owing to its high mineral density and molecular weight, primarily attributable to hydroxyapatite deposition, bone absorbs 30–40% more radiation energy than surrounding soft tissues [2]. Clinical studies have demonstrated that patients undergoing localized radiotherapy face substantially increased risks of radiation-induced bone complications, including pathological fracture, bone damage, and osteoradionecrosis. These complications manifest clinically as persistent pain, progressive functional impairment, and profound deterioration in health-related quality of life [3,4]. Concurrently, radiation-induced degradation of the bone matrix establishes a pro-metastatic microenvironment that promotes the seeding, colonization, and outgrowth of disseminated tumor cells in bone [5,6], thereby increasing the risks of skeletal metastasis and malignancy-associated hypercalcemia, affecting overall survival and health-related quality of life in cancer survivors. Consequently, radiotherapy-induced skeletal damage has emerged as an urgent issue in clinical radiation oncology. Currently, the therapeutic options for the prevention and management of radiation-induced bone injury are limited. Although anti-resorptive agents such as bisphosphonates are widely employed to attenuate radiation-associated bone loss, they neither halt nor reverse the underlying pathological cascade, including osteocyte dysfunction, vascular rarefaction, and matrix degradation. Moreover, prolonged administration carries well-documented risks of irreversible adverse effects, most notably medication-related osteonecrosis [7,8]. Furthermore, the absence of validated early biomarkers for radiation-induced bone injury means that by the time of therapeutic intervention, advanced, irreversible complications, such as osteoradionecrosis or pathological fracture, have already manifested, and the critical window for disease-modifying treatment has been missed [9]. Therefore, elucidating the molecular and cellular mechanisms underlying radiation-induced bone injury and developing novel, efficient bone protection strategies constitute pivotal priorities in both radiation oncology and skeletal biology.

Cellular senescence refers to stable, irreversible cell cycle arrest accompanied by the development of a pro-inflammatory secretory phenotype termed the senescence-associated secretory phenotype (SASP), and is now widely recognized as a key contributor to age-related bone loss [10] and radiation-induced tissue damage [11,12,13]. IR induces stress-induced premature senescence (SIPS) in multiple cell types within the bone microenvironment, characterized by marked upregulation of the cyclin-dependent kinase inhibitors P16^INK4a^ and P21^Cip1/Waf1^, as well as increased senescence-associated β-galactosidase (SA-β-gal) activity [14,15]. The progressive accumulation of senescent cells (SNCs) in irradiated bone tissue, coupled with their sustained and dysregulated SASP secretion, drives chronic inflammation and disrupts skeletal homeostasis. Although IR-induced dysfunction of osteoblasts [16] and osteoclasts [17] has been extensively characterized, accumulating evidence highlights osteocytes, the most abundant, mechanosensitive, and long-lived bone cells, as central regulators of the skeletal response to radiation injury [7,18]. Osteocytes serve as both primary mechanosensors and master regulators of bone remodeling. Their dysfunction or premature senescence disrupts the precise balance between bone formation and resorption, thereby predisposing individuals to osteopenia and osteoporosis [19]. Elucidating the molecular mechanisms driving IR-induced osteocyte senescence and identifying the regulatory pathways that mediate consequent bone homeostatic imbalance holds significant translational potential for developing targeted therapeutic strategies against post-radiotherapy skeletal injury.

Current evidence indicates that IR predominantly induces DNA double-strand breaks (DSBs), thereby activating the DNA damage response (DDR) pathway and initiating irreversible cell cycle arrest [9,20]. Beyond this classic DNA-centric mechanism, calcium ions (Ca^2+^) have emerged as a critical modulator of cellular senescence. As common intracellular second messengers, Ca^2+^ orchestrate diverse physiological processes, such as cell proliferation, differentiation, and apoptosis, through precisely regulated spatiotemporal dynamics [21,22]. Under normal conditions, intracellular Ca^2+^ levels are precisely maintained across key compartments, including the cytosol, mitochondria, and endoplasmic reticulum (ER). In contrast, pathological insults such as oxidative stress, DNA damage, and mechanical perturbation can disrupt this equilibrium, resulting in Ca^2+^ dyshomeostasis, characterized by elevated basal cytosolic Ca^2+^ concentration, mitochondrial Ca^2+^ overload, and ER Ca^2+^ depletion. Such dysregulation activates Ca^2+^-dependent effectors, including calpains, Ca^2+^/calmodulin-dependent protein kinase II (CaMKII), and the calcineurin/NFAT signaling axis, thereby directly or indirectly promoting cell cycle arrest and SASP expression. In bone tissue, Ca^2+^ signaling assumes particular functional significance, as osteocytes utilize mechanosensitive Ca^2+^ influx through channels such as PIEZO1 and TRPV4 to detect mechanical load and coordinate remodeling responses via paracrine and dendritic signaling [23]. Nevertheless, whether IR triggers Ca^2+^ overload specifically in osteocytes, and whether this perturbation functionally contributes to radiation-induced skeletal senescence, has not been investigated.

Recent studies in cancer biology have identified a calcium-dependent signaling axis centered on nuclear factor of activated T-cells, cytoplasmic 1 (NFATc1), and activating transcription factor 3 (ATF3) [24]. Dysregulated Ca^2+^ fluxes can influence cell cycle arrest and transcriptional programming of the SASP by activating downstream effectors, including Ca^2+^/calmodulin-dependent kinase II (CaMKII) and the calcineurin/NFAT signaling pathway. Notably, NFATc1, a master transcription factor induced by sustained intracellular Ca^2+^ elevation, has been implicated in inflammatory signaling, autophagy regulation, and senescence-associated phenotypes [25,26]. Concurrently, ATF3, a stress-inducible member of the AP-1/ATF/CREB transcription factor superfamily, functions as a key integrator of cellular stress responses to hypoxia, genotoxic injury, and oxidative stress [27,28]. ATF3 can modulate cell fate decisions such as proliferation, apoptosis, and differentiation, through homodimeric or heterodimeric DNA binding [29,30]. Critically, ATF3 serves as a functional node that links Ca^2+^ signaling and the DDR, modulating the onset, progression, and phenotypic severity of cellular senescence via chromatin remodeling, transcriptional regulation of cyclin-dependent kinase inhibitors (CDKIs), and selective control of SASP factor expression [31,32,33]. Potential crosstalk between ATF3 and NFAT family members has been reported, potentially involving transcriptional co-regulation or competitive binding at shared genomic loci [34]. These findings position the NFATc1-ATF3 axis as a central coordinator of Ca^2+^-dependent cell fate outcomes, encompassing senescence, apoptosis, and inflammatory amplification. Despite these advances, the involvement of Ca^2+^ overload and its downstream effectors, including NFATc1 and ATF3, in osteocyte-specific senescence following ionizing radiation remains uncharacterized.

Given that osteocytes express functional Ca^2+^-permeable ion channels and critically depend on spatiotemporally controlled Ca^2+^ signaling for mechanotransduction and paracrine regulation of bone remodeling, we hypothesized that ionizing radiation (IR) triggers pathological Ca^2+^ overload specifically in osteocytes, thereby activating the NFATc1-ATF3 signaling axis to drive cellular senescence and disrupt skeletal homeostasis. To test this hypothesis, we established an in vitro model of IR-induced osteocyte senescence using primary murine osteocytes isolated from calvarial bone fragments and exposed to 2 Gy X-ray irradiation. We pharmacologically modulated the intracellular Ca^2+^ dynamics using the cell-permeable Ca^2+^ chelator BAPTA-AM and the L-type calcium channel blocker verapamil to determine the causal necessity of Ca^2+^ overload in this process. We assessed cell proliferation, morphological changes, the persistent DNA damage response, canonical senescence markers, and intracellular calcium levels; quantified SASP factor secretion; and functionally evaluated the osteogenic and osteoclastogenic differentiation potentials of bone marrow stromal cells (BMSCs) and bone marrow-derived macrophages (BMMs) co-cultured with osteocyte-conditioned media, and the activation status of the NFATc1-ATF3 cascade. Collectively, these investigations are expected to provide mechanistic insight into Ca^2+^-mediated osteocyte dysfunction following IR exposure and identify the NFATc1-ATF3 axis as a therapeutically targetable node for preventing or ameliorating IR-induced skeletal senescence and associated bone fragility.

## 2. Materials and Methods

### 2.1. Isolation of Primary Murine Osteocytes and Experimental Group

#### 2.1.1. Isolation and Culture of Primary Murine Osteocytes

Primary murine osteocytes (OCYs) were isolated from the calvaria bones of 6-week-old BALB/c mice using a modified enzymatic digestion protocol adapted from established methods [35]. Briefly, calvaria were obtained following aseptic dissection and removal of adherent soft tissues and marrow contents. Sequential enzymatic digestion was performed at 37 °C with agitation, starting with 0.25% Trypsin solution (25200072; Gibco, Waltham, MA, USA), followed by three rounds of digestion with 0.1% (*w*/*v*) collagenase type II (C6885; Sigma-Aldrich, St. Louis, MO, USA) and 0.05 mM EDTA (E1170; Solarbio, Beijing, China), and finally with 0.1% (*w*/*v*) collagenase type I (C0130; Sigma-Aldrich, St. Louis, MO, USA). Then, the bone fragments and the liberated cell suspension were cultured in α-MEM (C12571500BT; Gibco, Waltham, MA, USA) supplemented with 10% FBS (10099141C; Gibco, Waltham, MA, USA). The cells were plated and cultured at 37 °C under 5% CO_2_. After 7 days, dendritic cells exhibiting characteristic stellate morphology and dendritic processes were selectively harvested and further characterized by morphology, ALP staining, and E11/gp38 expression. All functional assays were conducted using cells from passages 2 to 3.

#### 2.1.2. Identification of Primary Murine Osteocytes

##### Morphological Observation

Cellular morphology was observed under an inverted phase-contrast microscope (Leica DMI3000; Leica Microsystems, Wetzlar, Germany). Primary OCYs and osteoblasts (OBs) were compared for morphological identification.

##### Alkaline Phosphatase (ALP) Staining

The primary OCYs and OBs were seeded into 6-well plates. When cell confluence reached 80%, ALP staining was performed. Then, the culture medium was removed, and the cells were washed once with PBS. The cells were then fixed with 2.5% glutaraldehyde (30092436, Sinopharm Chemical Reagent Co., Ltd., Shanghai, China) for 5 min at room temperature in the dark. After fixation, the staining working solution was prepared using a BCIP/NBT Alkaline Phosphatase Color Development Kit (C3206; Beyotime, Shanghai, China). Then, 1 mL of the working solution was added to each well, and the cells were incubated in a 37 °C water bath in the dark for 30 min. After incubation, the cells were washed three times with PBS (G4202; Servicebio, Wuhan, China) to terminate the reaction. The stained cells were observed and imaged under a Nikon ECLIPSE 80i microscope (Nikon Instruments Inc., Melville, NY, USA).

##### Detection of the Osteocyte-Specific Marker E11

The primary OCYs and OBs were seeded into 6-well plates and cultured to 80% confluence. The protein expression level of the osteocyte-specific marker E11/gp38 was assessed by Western blot. Detailed procedures are described in Section 2.9.

#### 2.1.3. Experimental Design and Treatment Groups

To evaluate the functional contribution of calcium dyshomeostasis to IR-induced osteocyte senescence, primary murine osteocytes in the logarithmic growth phase were seeded in 6-well plates and cultured for 24 h. The cells were randomized into four experimental groups and subjected to the following interventions.

(1) Sham control (CON): Cells underwent sham irradiation (identical handling without X-ray activation) and were cultured for 72 h. The sham-irradiated controls underwent identical handling, including plate removal from the incubator, placement in the irradiator chamber, and duration of exposure without activation of the X-ray source.
(2) Ionizing radiation (IR): Cells received a single 2 Gy X-ray dose and were cultured for 72 h.
(3) IR+BAPTA-AM: Cells were pre-incubated with 10 μM BAPTA-AM (T6245; TargetMol Chemicals Inc., Shanghai, China) for 30 min prior to irradiation, then exposed to 2 Gy and cultured for 72 h.
(4) IR+Verapamil: Cells were pre-incubated with 10 μM verapamil (HY-14275; MedChemExpress, Monmouth Junction, NJ, USA) for 4 h prior to irradiation, then exposed to 2 Gy and cultured for 72 h. ST038

Cells were irradiated with a single dose of 2 Gy X-rays using an X-Rad 320 Biological Irradiator (PXi, Houston, TX, USA). The irradiation parameters were strictly controlled: tube voltage of 320 kV, tube current of 4.3 mA, source-to-surface distance of 50.0 cm, and dose rate of 102.3 cGy/min. All pharmacological agents were dissolved in DMSO (ST038; Beyotime, Shanghai, China) at stock concentrations, ensuring a final vehicle concentration ≤0.1% (*v*/*v*). The culture medium was replaced immediately after irradiation or sham treatment to remove residual compounds.

### 2.2. Cell Biological Function Assays

#### 2.2.1. Assessment of Cellular Proliferation via EdU Incorporation

Proliferation activity was quantified using the 5-ethynyl-2′-deoxyuridine (EdU) incorporation kit (C0071S; Beyotime, Shanghai, China). Primary murine osteocytes were seeded on coverslips in 24-well plates and allowed to adhere for 24 h. Subsequently, cells were incubated with 25 μM EdU for 2 h at 37 °C under 5% CO_2_. Following fixation with 4% paraformaldehyde (PFA, P1110; Solarbio, Beijing, China) for 15 min and permeabilization with 0.3% Triton X-100 for 10 min at room temperature, EdU-labeled DNA was visualized via copper-catalyzed azide–alkyne cycloaddition (click reaction) using the supplied reagents. Nuclei were counterstained with DAPI (D523; Dojindo, Kumamoto, Japan) for 5 min. The coverslips were mounted with anti-fade mounting medium and imaged using a Nikon Ni-U compound fluorescence microscope (Nikon Instruments Inc., Melville, NY, USA) at 200× magnification. For each well, 10 non-overlapping fields of view were randomly selected and acquired under identical exposure settings. EdU-positive nuclei (green) and total nuclei (blue) were enumerated using the ImageJ software (Version 1.54p; NIH, Bethesda, MD, USA), and the proliferation index was calculated as follows: EdU-positive rate (%) = (number of EdU-positive nuclei/total number of DAPI-stained nuclei) × 100.

#### 2.2.2. Quantification of Cellular Viability via CCK-8 Assays

Cellular viability was assessed using the Cell Counting Kit-8 (CK04; Dojindo, Kumamoto, Japan) according to the manufacturer’s protocol with minor modifications for primary osteocyte culture. The primary murine osteocytes were seeded in 96-well plates at a density of 3 × 10^3^ cells per well and allowed to adhere for 24 h prior to treatment initiation. At 72 h post-irradiation, culture medium was carefully aspirated, and 110 µL of freshly prepared CCK-8 working solution (10 µL of CCK-8 stock solution diluted in 100 µL of complete culture medium) was added to each well. The plates were incubated for 2 h at 37 °C under 5% CO_2_ in the dark to prevent reagent photodegradation. Absorbance at 450 nm was measured with a microplate reader (Epoch^TM^; Biotech, Vicenza, Italy). Cellular viability was expressed as the raw absorbance (OD) values.

#### 2.2.3. Flow Cytometric Analysis of Cell Cycle Distribution

Cell cycle distribution was analyzed by flow cytometry with the Cell Cycle Staining Kit (70-CCS012; Lianke Biotech, Hangzhou, China). The primary murine osteocytes were harvested via gentle trypsin–EDTA dissociation and fixed in 70% ethanol overnight at −20 °C. The fixed cells were washed with PBS, resuspended in 1 mL of DNA staining solution containing 50 μg/mL PI and 100 μg/mL RNase A, and incubated for 30 min at room temperature in the dark. Prior to acquisition, samples were filtered through a 40-μm nylon mesh to remove aggregates. DNA content was analyzed on a CytoFLEX S flow cytometer (Beckman Coulter, Inc., Fullerton, CA, USA). Cell cycle phase distribution was analyzed using the ModFit LT software (Version 5.0; Verity Software House, Topsham, ME, USA).

#### 2.2.4. Flow Cytometric Analysis of Apoptosis

Apoptosis was analyzed by flow cytometry with the Annexin V-FITC/PI Apoptosis Detection Kit (AP101-30; Lianke Biotech, Hangzhou, China). Cells were harvested via gentle trypsin–EDTA dissociation, washed with PBS, and stained with 5 μL of Annexin V-FITC and 10 μL of propidium iodide (PI) according to the manufacturer’s instructions. After incubation for 30 min at room temperature in the dark, the stained cells were analyzed on a CytoFLEX S flow cytometer. Data were acquired and processed using the FlowJo software (Version 10.8.1; BD Biosciences, Franklin Lakes, NJ, USA). The apoptosis rate was calculated as the percentage of Annexin V-positive cells, including both early and late apoptotic populations, using the following formula: apoptosis rate (%) = (number of apoptotic cells/total number of cells) × 100%.

#### 2.2.5. Immunofluorescence-Based Morphological Analysis of Osteocyte Morphology

To characterize radiation-induced morphological alterations, including dendritic arborization, cell body enlargement, and cytoskeletal reorganization, immunofluorescence staining was performed to visualize F-actin and nuclear architecture. Primary murine osteocytes were seeded onto 3.5 cm glass-bottom dishes at a density of 1 × 10^5^ cells per dish and allowed to adhere for 24 h. Following exposure to 2 Gy X-ray irradiation, the cells were cultured for an additional 24 h to allow phenotypic manifestation. The cells were washed twice with PBS following aspiration of the culture medium. Fixation was performed with 2.5% glutaraldehyde for 10 min, followed by two additional washes with PBS. F-actin was labeled with tetramethylrhodamine (TRITC)-conjugated phalloidin (diluted 1:500; CA1610; Solarbio, Beijing, China) for 1 h, and nuclei were counterstained with DAPI (diluted 1:500) for 30 s in the dark. Following three PBS washes, samples were mounted with anti-fade mounting medium. Images were captured using a Nikon Ni-U compound fluorescence microscope at 200× magnification under identical acquisition settings for all groups. Dendritic length was measured using the ImageJ software across 10 non-overlapping fields per well.

### 2.3. DNA Damage Response Assessment

γ-H2AX foci formation in OCYs was assessed by immunofluorescence microscopy to evaluate persistent DNA damage response signaling following irradiation. OCYs were seeded uniformly onto 3.5 cm glass coverslips at a density of 1 × 10^5^ cells per well. After 24 h of culture, the cells were irradiated with 2 Gy X-rays and subsequently incubated for an additional 24 h prior to γ-H2AX immunostaining. The cells were then washed twice with PBS and fixed with 4% PFA for 20 min at room temperature. Nonspecific binding was blocked with QuickBlock™ immunostaining blocking solution (P0260; Beyotime, Shanghai, China) for 1 h. Primary antibody against γ-H2AX (ab81299; Abcam, Cambridge, MA, USA, rabbit monoclonal, 1:500 dilution) was applied overnight at 4 °C. Subsequently, the cells were incubated with FITC-conjugated goat anti-rabbit IgG secondary antibody (A0562; Beyotime, Shanghai, China) for 2 h and counterstained with DAPI (1:500 dilution) at room temperature in the dark. Coverslips were mounted with anti-fade mounting medium and imaged using a Nikon Ni-U compound fluorescence microscope. For quantification, ten non-overlapping fields of view were randomly selected per coverslip. A cell was scored as γ-H2AX-positive if it exhibited ≥10 distinct, well-resolved nuclear foci. The percentage of γ-H2AX-positive cells was calculated as follows: γ-H2AX-positive cells (%) = (number of γ-H2AX-positive cells/total number of DAPI-stained nuclei) × 100%.

### 2.4. Characteristic Senescence Phenotype Assays

To reliably identify irradiation-induced osteocyte senescence, a multiparametric assessment strategy was adopted. The detailed procedures for each assay are described in the following subsections.

#### 2.4.1. Senescence-Associated β-Galactosidase (SA-β-gal) Activity Assay

Senescence-associated β-galactosidase (SA-β-gal) activity was assessed as a senescence-associated marker using a kit (C0602; Beyotime, Shanghai, China), following the manufacturer’s instructions. OCYs were seeded at a density of 1 × 10^5^ cells per well in 6-well plates and allowed to adhere for 24 h under standard culture conditions. Thereafter, cells were irradiated with 2 Gy X-rays and maintained for an additional 72 h. The cells were rinsed twice with PBS, fixed with the provided β-galactosidase fixative solution for 15 min at room temperature, and subsequently incubated with SA-β-gal staining solution (pH 6.0) at 37 °C overnight in a dry incubator. Following staining, the cells were washed three times and imaged using a microscope. For quantitative analysis, 200 cells per well were randomly selected. The percentage of SA-β-gal-positive (blue-stained) cells was calculated as follows: number of SA-β-gal-positive cells (%) = (number of SA-β-gal-positive cells/total number of cells) × 100%.

#### 2.4.2. Senescence-Associated Chromatin Condensation-like Features Assessment

To assess nuclear chromatin condensation-like morphology associated with cellular senescence, osteocytes (OCYs) were seeded at a density of 1 × 10^5^ cells per well onto 3.5 cm glass coverslips placed in 6-well plates. Following 24 h of adherence, the cells were irradiated with 2 Gy X-rays and incubated for an additional 24 h prior to DAPI staining. The culture medium was carefully discarded, and the cells were washed twice with PBS. Fixation was performed with 4% PFA for 15 min, followed by two washes with PBS. The cells were then incubated with DAPI (1:500 dilution) for 5 min in the dark. Images were captured using a Nikon Ni-U compound fluorescence microscope. For quantitative analysis, ten non-overlapping fields of view were randomly selected per coverslip. Cells exhibiting condensed punctate DAPI-dense nuclear foci distinct from diffuse chromatin staining were quantified as cells displaying chromatin condensation-like features. This DAPI-based assessment was used as a supportive morphological readout and was not considered definitive validation of genuine senescence-associated heterochromatin foci (SAHF) formation. The percentage of DAPI-dense foci-positive cells was calculated as follows: DAPI-dense foci-positive cells (%) = (number of positive cells/total number of cells) × 100%.

#### 2.4.3. Detection of Key Senescence-Associated Proteins

Osteocytes were seeded in 6-well plates at a density of 1 × 10^5^ cells per well. After 24 h of adherence, the cells were irradiated. The mRNA and protein expression levels of p16 and p21, two key genes involved in senescence-associated pathways, were examined by RT-qPCR and Western blot, respectively. The detailed procedures are provided in Section 2.9 and Section 2.10.

#### 2.4.4. Detection of SASP Markers

Osteocytes were seeded in T25 flasks at a density of 2.5 × 10^5^ cells per flask. After 24 h of adherence, the cells were irradiated with 2 Gy X-rays and subsequently cultured for an additional 24 h. Thereafter, the complete culture medium was replaced with serum-free medium, and an additional 24 h incubation of the cells was performed to allow accumulation of secreted factors. The conditioned medium was collected and stored at −80 °C until analysis. SASP factor secretion was assessed using the Mouse XL Cytokine Array kit (ARY028; R&D Systems, Inc., Minneapolis, MN, USA). Briefly, all reagents were equilibrated to room temperature for 30 min prior to use. After blocking with blocking reagent on a shaker for 1 h, the membrane was washed three times with wash buffer, and then incubated overnight at 4 °C with cell culture supernatants pre-mixed with biotinylated detection antibodies. After three washes, the membrane was incubated for 1 h with streptavidin–HRP conjugate (diluted 1:100 in assay diluent to yield a 1× working solution). After three final washes, chemiluminescent detection reagents (Solution 1 and Solution 2, mixed 1:1 *v*/*v*) were applied for 1 min, and signals were captured using an imaging system. Spot intensities were quantified by pixel-density analysis, and SASP factor levels were normalized to internal positive controls on each membrane.

### 2.5. Assessment of Intracellular Calcium Levels

Intracellular calcium levels were quantified using the Fluo-4 AM calcium indicator (S1061S; Beyotime, Shanghai, China). Briefly, OCYs were seeded in T25 flasks at a density of 2.5 × 10^5^ cells per flask and allowed to adhere for 24 h. For time-course analysis, cells were irradiated with 2 Gy X-rays and collected at 0.5, 6, 24, 48, and 72 h post-irradiation. For dose–response analysis, cells were exposed to 0.5, 1, 2, or 4 Gy X-rays and collected at 6 h post-irradiation. To assess the effects of calcium-modulating interventions on irradiation-induced calcium accumulation, cells were pretreated with BAPTA-AM or verapamil before 2 Gy irradiation and analyzed at 6 h post-irradiation. Prior to measurement, the cells were washed with pre-warmed PBS, incubated with Fluo-4 AM working solution for 30 min at 37 °C in the dark, and subsequently washed three times with PBS to remove excess dye. Fluorescence intensity, which reflects intracellular Ca^2+^ levels, was measured immediately by flow cytometry.

### 2.6. Co-Culture Differentiation Assays

#### 2.6.1. Collection of Osteocyte-Conditioned Medium

OCYs were seeded in T75 culture flasks and allowed to adhere for 24 h. The cells were then irradiated with 2 Gy X-rays and cultured for an additional 24 h. Thereafter, the culture medium was aspirated, and the cells were washed with PBS to remove residual serum and debris. Fresh serum-free medium was added, and the cells were incubated for another 24 h to promote the secretion of soluble factors. The conditioned medium was collected, centrifuged at 300× *g* for 5 min, filtered through a 0.22 µm sterile syringe filter, aliquoted into single-use vials, and stored at −80 °C. Conditioned media from the non-irradiated control, irradiated, irradiated + BAPTA-AM-treated, and irradiated + verapamil-treated groups were designated as CON-CM, IR-CM, IR+BAP-CM, and IR+Ver-CM, respectively, and used for downstream SASP profiling and co-culture differentiation assays.

#### 2.6.2. Co-Culture Experiments with BMSCs

##### Isolation of BMSCs

Primary bone-marrow-derived mesenchymal stem cells (BMSCs) were isolated from the femurs and tibiae of 3-week-old male Sprague-Dawley (SD) rats using the whole bone marrow adherent method. The rats were humanely euthanized by intraperitoneal injection of 2% sodium pentobarbital, followed by surface disinfection via full-body immersion in 75% ethanol for disinfection. Under strict sterile conditions, the femurs and tibiae were aseptically excised, and residual muscles, tendons, and connective tissues were meticulously removed using sterile gauze and surgical scissors. Both epiphyseal ends of each bone were transected with sterile bone cutters to fully expose the marrow cavity. The cavity was then flushed repeatedly with α-MEM (supplemented with 10% FBS and 1% penicillin–streptomycin) using a 1 mL syringe until the cortical shaft appeared translucent white. The collected bone marrow suspension was centrifuged, resuspended in complete α-MEM, filtered through a cell strainer, and seeded into T25 culture flasks. After 48 h, non-adherent hematopoietic cells were removed by gentle medium replacement, and the remaining adherent cells were washed with PBS to eliminate residual debris. Fresh complete medium was added, and the cultures were maintained for 5–7 days. Adherent, fibroblast-like cells exhibiting characteristic spindle morphology were designated as passage 0 (P0) BMSCs. At 80% confluence, the cells were dissociated with 0.25% trypsin–EDTA. Trypsinization was neutralized with serum-containing medium, and the cells were collected via centrifugation before replating for expansion and subsequent co-culture experiments.

##### Colony-Forming Unit (CFU) Assay

BMSCs were seeded in 6 cm culture dishes at a density of 2 × 10^3^ cells per dish and allowed to adhere for 24 h. Thereafter, the medium was replaced with α-MEM supplemented with 50% conditioned medium (CM), specifically, control CM (CON-CM), irradiation-induced CM (IR-CM), IR co-treated with BAPTA-AM CM (IR+BAP-CM), or IR co-treated with verapamil CM (IR+Ver-CM). The medium was refreshed every other day over the 14-day culture duration. At the endpoint, the medium was carefully aspirated, followed by fixation of adherent cells with methanol at room temperature for 15 min. The cells were then stained with crystal violet solution for 10 min in the dark. Unbound dye was thoroughly rinsed off with distilled water, and colonies comprising ≥50 morphologically intact, densely packed cells were enumerated under a Nikon ECLIPSE 80i optical microscope. The colony-forming efficiency was calculated as the number of colonies and compared across experimental groups.

##### Osteogenic Differentiation Potential Assay

To evaluate the impact of irradiated osteocyte-derived conditioned medium (CM) on the osteogenic differentiation potential of BMSCs, Passage 3 BMSCs were seeded in 24-well plates at a density of 3 × 10^4^ cells per well. At 24 h post-seeding, the cells were switched to osteogenic induction medium consisting of α-MEM supplemented with 15% FBS, 10 mM β-glycerophosphate (50020; Sigma-Aldrich, St. Louis, MO, USA), 10 nM dexamethasone (ST1254; Beyotime, Shanghai, China), 50 µg/mL ascorbic acid (BS247; Beijing Lanjiake Technology Co., Ltd., Beijing, China), and 50% CM (CON-CM, IR-CM, IR+BAP-CM, or IR+Ver-CM). The induction medium was refreshed every 48 h. The osteogenic differentiation capacity was quantitatively evaluated by an alkaline phosphatase (ALP) activity assay and qualitatively confirmed by Alizarin Red S staining for extracellular calcium deposition.

1. ALP activity assay: Following 7 days of osteogenic induction, cells were washed three times with PBS (5 min each) after removal of the culture medium. The cells were then fixed with 2.5% glutaraldehyde for 5 min under light-protected conditions, followed by three gentle rinses with PBS (3 min each). ALP staining was performed using the BCIP/NBT Alkaline Phosphatase Color Development Kit. Following staining, images were acquired under a Nikon ECLIPSE 80i optical microscope. The percentage of ALP-positive area was quantified using the ImageJ software (NIH, Bethesda, MD, USA) by thresholding-stained regions across 10 non-overlapping fields per well.
2. Mineralization capacity assay: Following 21 days of osteogenic induction, mineralized nodule formation was assessed by Alizarin Red S (ARS) staining. Briefly, cells were fixed using 4% PFA for 30 min, followed by three rinses with PBS to ensure complete removal of residual fixative. ARS staining solution (ALIR-10001; Cyagen Biosciences, Guangzhou, China) was added and incubated for 7 min in the dark. Unbound dye was removed by extensive washing with PBS. Images were acquired under a Nikon ECLIPSE 80i microscope. The percentage of ARS-positive area was quantified using the ImageJ software by applying a consistent intensity threshold across all samples, based on 10 non-overlapping fields per well.

#### 2.6.3. Co-Culture Experiments with BMMs

##### Isolation of BMMs

Primary bone marrow-derived macrophages (BMMs) were isolated from the femurs and tibiae of 6-week-old male BALB/c mice using density gradient centrifugation. The mice were humanely euthanized by intraperitoneal injection of an overdose of sodium pentobarbital, followed by cervical dislocation to ensure death. The entire carcass was immersed in 75% ethanol for surface disinfection. Under sterile laminar flow conditions, the femurs and tibiae were aseptically dissected, and adherent muscle, tendons, and connective tissues were gently removed using sterile forceps and gauze. After carefully excising both epiphyseal ends, the bone marrow was flushed with α-MEM, and the cell suspension was filtered through a 70 μm cell strainer to remove bone fragments and clumps. The filtrate was layered onto the surface of lymphocyte separation medium (17544602; Cytiva, Dorset, UK). After centrifugation at 800× *g* for 30 min at 20 °C, the cloudy middle layer (containing mononuclear cells) was carefully aspirated into a new tube. The cells were washed twice with cold PBS to remove residual separation medium. The final pellet was resuspended in complete culture medium and seeded into T25 flasks. After 24 h incubation, non-adherent cells were collected, centrifuged at 300× *g* for 5 min, and resuspended in complete culture medium containing 25 ng/mL recombinant murine macrophage colony-stimulating factor (M-CSF, 315-02; PeproTech, Cranbury, NJ, USA). The cells were seeded at 3 × 10^3^ cells/well in 96-well plates and 5 × 10^5^ cells/well in 6-well plates for subsequent osteoclast differentiation assays.

##### Tartrate-Resistant Acid Phosphatase (TRAP) Staining

To assess osteoclast differentiation, BMMs seeded in 96-well plates were cultured in osteoclast induction medium consisting of α-MEM supplemented with 25 ng/mL M-CSF, 25 ng/mL recombinant murine RANKL (315-11; PeproTech, Rocky Hill, NJ, USA), and 50% conditioned medium (CON-CM, IR-CM, IR+BAP-CM, or IR+Ver-CM). The cells were incubated for 5 days with the medium refreshed every 48 h. TRAP staining was performed using the TRAP kit (387A-1KT; Sigma-Aldrich, St. Louis, MO, USA). Briefly, cells were fixed with 2.5% glutaraldehyde for 5 min, rinsed gently three times with PBS, then incubated with TRAP working solution for 1 h at 37 °C in the dark using a pre-equilibrated water bath. Images were acquired under a microscope. Osteoclasts were defined as TRAP-positive multinucleated cells containing ≥3 nuclei, and the number of osteoclasts per well was quantified by counting across eight non-overlapping fields per well.

##### Osteoclastogenic Function-Related Gene Expression

BMMs seeded in 6-well plates were cultured in osteoclast induction medium (α-MEM supplemented with 25 ng/mL M-CSF, 25 ng/mL RANKL, and 50% conditioned medium) for 5 days, and total RNA was extracted from the harvested cells. RT-qPCR was performed to quantify mRNA expression levels of osteoclast function-related genes, including osteoclast-associated receptor (*Oscar*) and osteoclast stimulatory transmembrane protein (*OC-stamp*). The detailed experimental methods are described in the following section.

### 2.7. Subcellular Fractionation for NFATc1 Nuclear Translocation

To evaluate NFATc1 nuclear translocation, immunofluorescence staining was performed. Cells seeded on sterile glass coverslips were fixed with 4% PFA for 10 min, permeabilized with 0.1% Triton X-100 for 5 min, and blocked with 5% bovine serum albumin (BSA; ST025; Beyotime, Shanghai, China) for 1 h. After overnight incubation at 4 °C with rabbit polyclonal anti-NFATc1 antibody (1:250 dilution; ab25916; Abcam, Cambridge, MA, USA), the cells were incubated with FITC-conjugated goat anti-rabbit IgG secondary antibody (1:500 dilution) for 1 h at room temperature in the dark. The nuclei were counterstained with DAPI for 5 min. Coverslips were mounted onto slides with anti-fade mounting medium and sealed. Images were captured using a Nikon Ni-U compound fluorescence microscope. For quantification, nine non-overlapping fields per coverslip were captured under identical acquisition parameters. Nuclear and cytoplasmic NFATc1 fluorescence intensities were measured using the Image J software, and the nuclear-to-cytoplasmic fluorescence intensity ratio was calculated for ≥100 cells per condition. Additionally, the percentage of cells exhibiting clear nuclear enrichment of NFATc1 (defined as N/C ratio ≥ 0.5) was quantified.

### 2.8. RNA Extraction and Quantitative Real-Time PCR

Total RNA was extracted from cultured cells using the Simply P Total RNA Extraction Kit (BSC52S1; Bioflux, Beijing, China), following the manufacturer’s protocol. The quantity and quality of the extracted total RNA were evaluated by spectrophotometric analysis at 260 nm and the A260/A280 absorbance ratio, respectively. Complementary DNA (cDNA) was synthesized from 1 μg of DNase I-treated total RNA using the FastKing gDNA Dispelling RT SuperMix (KR118-02; Tiangen Biotech, Beijing, China). Quantitative real-time PCR (qRT-PCR) was performed on an ABI QuantStudio 5 system (Applied Biosystems, Carlsbad, CA, USA) using PowerUp SYBR Green Master Mix (Invitrogen; Thermo Fisher Scientific, Waltham, MA, USA). Relative gene expression was calculated using the 2^−ΔΔCt^ method and normalized to *Gapdh*. The following primer sequences were used (Table 1).

### 2.9. Western Blotting Analysis

Cultured cells were lysed using RIPA lysis buffer (P0013B; Beyotime, Shanghai, China) supplemented with freshly prepared protease and phosphatase inhibitor cocktails (ST506; Beyotime, Shanghai, China). After lysis, the cell lysates were collected and centrifuged at 12,000 rpm for 15 min at 4 °C. The resulting supernatants were then harvested as the total protein fractions. Protein concentration was quantified using the BCA Protein Assay Kit (P0012; Beyotime, Shanghai, China). Equal amounts of protein (30 μg per lane) were resolved by SDS-polyacrylamide gel electrophoresis on 10% resolving gels under a constant voltage of 80 V for 30 min, followed by 120 V for 30 min. The separated proteins were then transferred onto 0.2 µm polyvinylidene fluoride (PVDF) membranes (ISEQ00010; Millipore, Billerica, MA, USA) using a semi-dry transfer apparatus at a constant current of 300 mA for 60 min. The membranes were blocked for 1 h with 5% non-fat milk, then incubated overnight at 4 °C with primary antibodies with gentle agitation. Following three washes with TBST, the membranes were incubated with horseradish peroxidase (HRP)-conjugated secondary antibodies (SA00001-2; SA00001-1; Proteintech, Wuhan, China) for 1 h at room temperature with gentle agitation. Immunoreactive bands were detected by enhanced chemiluminescence (ECL) substrate (D3308; Beyotime, Shanghai, China) and captured on the chemiluminescence imaging system (Tanon 4600, Tanon Life Science Co., Ltd., Shanghai, China). Band intensities were quantified using the ImageJ software, and target protein expression levels were normalized to β-actin. Only the band at the expected molecular weight was used for densitometric analysis (Table 2).

### 2.10. Statistical Analysis

All experiments were performed independently at least three times, with technical replicates indicated in the corresponding figure legends. Data are presented as mean ± standard deviation (SD). Statistical analysis was conducted using the GraphPad Prism 10.0 software (GraphPad Software, San Diego, CA, USA). Normal distribution and homogeneity of variance were assessed by the Shapiro–Wilk test (α = 0.05) and Levene’s test (α = 0.05) before selecting inferential tests. When assumptions were met, comparisons between two groups were conducted using the unpaired, two-tailed Student’s *t*-test; multi-group comparisons employed one-way ANOVA followed by Tukey’s post hoc test. When either assumption was violated, non-parametric alternatives were applied: the Mann–Whitney U test for two-group comparisons and the Kruskal–Wallis test with Dunn’s post hoc correction (accounting for multiple comparisons) for multi-group analyses. Statistical significance was defined as * *p* < 0.05, ** *p* < 0.01, *** *p* < 0.001. The exact sample size (n) for each experiment, defined strictly as representing the number of independent biological replicates, is now uniformly reported in every relevant figure legend.

## 3. Results

### 3.1. Ionizing Radiation Induces Function Impairment in Osteocytes

**Figure 1:**
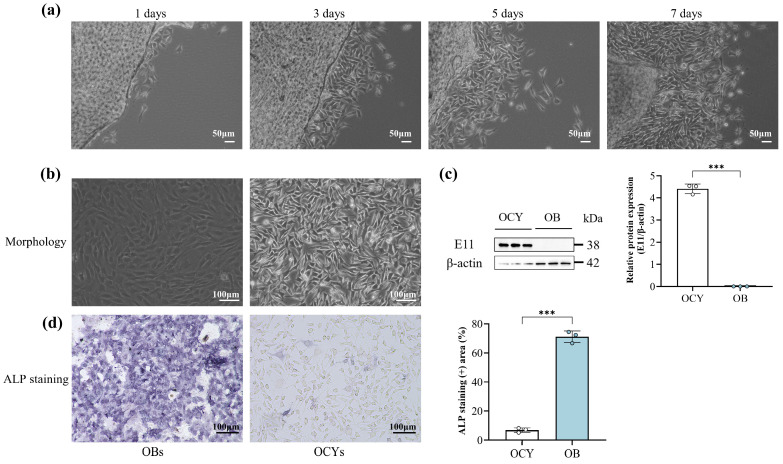
Isolation, culture and identification of primary OCYs. (**a**) Representative phase-contrast images of primary OCYs on days 1, 3, 5, and 7 after isolation (100× magnification, scale bar = 50 µm); (**b**) representative morphology of primary OCYs and primary OBs (100× magnification, scale bar = 100 µm); (**c**) Western blot analysis of the osteocyte-specific marker E11/gp38 in primary OCYs and OBs; (**d**) representative alkaline phosphatase (ALP) staining of primary OCYs and primary OBs (100× magnification, scale bar = 100 µm); and quantitative analysis of ALP-positive area. *** *p* < 0.001.

Primary osteocytes (OCYs) were isolated from mouse calvaria using a modified sequential digestion method. As shown in Figure 1a, scattered cells were observed around the bone fragments after 1 day of culture. After 3 days, cells began to migrate and proliferate, gradually displaying stellate morphology with dendritic processes. When cell density around the bone fragments reached 80–90%, cells were collected for ALP staining and detection of characteristic marker proteins. Compared with primary osteoblasts (OBs), OCYs exhibited a characteristic dendritic morphology and also showed higher protein expression of the osteocyte-specific marker E11/gp38 than OBs (Figure 1b,c). Moreover, OCYs exhibited markedly lower ALP activity, as evidenced by significantly fewer ALP-positive cells (Figure 1d). These morphological, histochemical, and molecular characteristics support successful enrichment of primary OCYs for subsequent experiments by the modified sequential digestion method.

**Figure 2:**
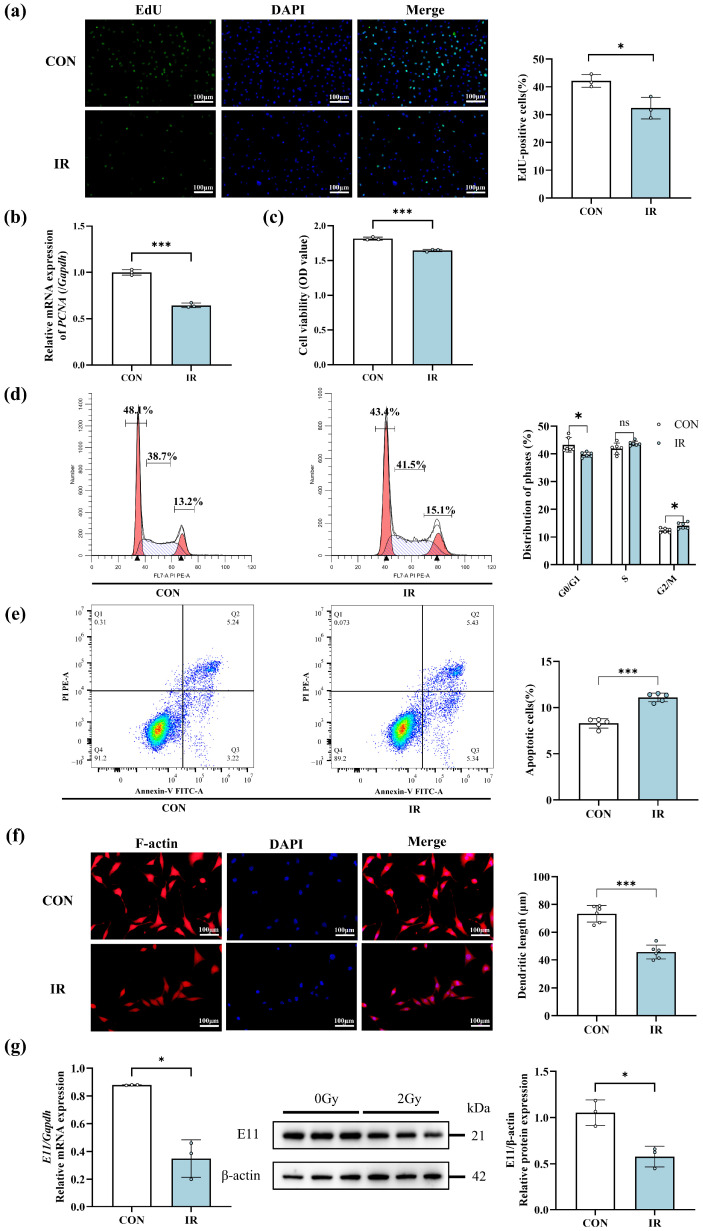
Ionizing radiation induces multifaceted functional impairment in primary osteocytes. (**a**) Representative EdU staining images (100× magnification, scale bar = 100 µm) and quantification of proliferating osteocytes at 24 h after 2 Gy X-ray irradiation; (**b**) relative mRNA expression of proliferating cell nuclear antigen *PCNA*) in irradiated versus sham-irradiated osteocytes at 24 h post-irradiation (n = 3); (**c**) cellular viability assessed by CCK-8 assay at 72 h post-irradiation (n = 3); (**d**) flow cytometry-based cell cycle distribution and quantitative analysis showing G2/M phase accumulation in irradiated osteocytes at 72 h post-irradiation (n = 6); (**e**) flow cytometry-based apoptotic rates and quantitative analysis in irradiated osteocytes at 72 h post-irradiation (n = 6); (**f**) F-actin immunofluorescence staining revealing dendritic retraction and somatic enlargement (100× magnification, scale bar = 100 µm) with corresponding morphometric quantification of average dendritic length (n = 6); (**g**) relative mRNA and protein expression levels of E11/gp38 in irradiated osteocytes. * *p* < 0.05, *** *p* < 0.001.

To investigate the effects of ionizing radiation on osteocyte morphology, proliferation, and viability, primary osteocytes were exposed to 2 Gy X-ray irradiation. The EdU incorporation assay revealed that 2 Gy irradiation significantly suppressed OCY proliferative capacity: the proportion of EdU-positive cells decreased by approximately 23.2% at 24 h after irradiation relative to the sham-irradiated controls (*p* < 0.05, Figure 2a). Consistent with this, mRNA expression of proliferating cell nuclear antigen (*PCNA*), a canonical marker of active DNA replication and cell proliferation, was reduced by 35.6% in irradiated osteocytes compared with controls (*p* < 0.001, Figure 2b). Similarly, the CCK-8 assay demonstrated a significant 9.36% reduction in relative viability in irradiated osteocytes compared with controls (*p* < 0.001, Figure 2c). Collectively, these complementary assays indicate that 2 Gy X-ray irradiation robustly impairs both proliferative capacity and cellular viability in osteocytes. Flow cytometric cell cycle analysis further showed that irradiation triggered G2/M phase arrest (*p* < 0.001, Figure 2d), indicating DNA damage-induced checkpoint activation. Annexin V/PI flow cytometric analysis demonstrated a measurable increase in apoptotic cells following irradiation, with apoptotic rates increasing from approximately 8.3% in control cells to 11.1% after 2 Gy irradiation (*p* < 0.001, Figure 2e). Moreover, F-actin fluorescence staining revealed substantial morphological disruption, evidenced by marked dendritic shortening. Quantitative morphometric analysis confirmed a 37.6% reduction in average dendrite length following irradiation (*p* < 0.001, Figure 2f). Critically, both the mRNA and protein levels of E11/gp38, an osteocyte-associated protein involved in dendritic architecture and cytoskeletal organization, were significantly downregulated (*p* < 0.05, Figure 2g). Collectively, these findings demonstrate that 2 Gy X-ray irradiation induces multifaceted functional impairment in osteocytes, encompassing suppressed proliferation, diminished viability, G2/M cell cycle arrest, limited apoptosis, and dendritic retraction.

### 3.2. Ionizing Radiation Triggers Persistent DNA Damage and Premature Senescence in Osteocytes

**Figure 3:**
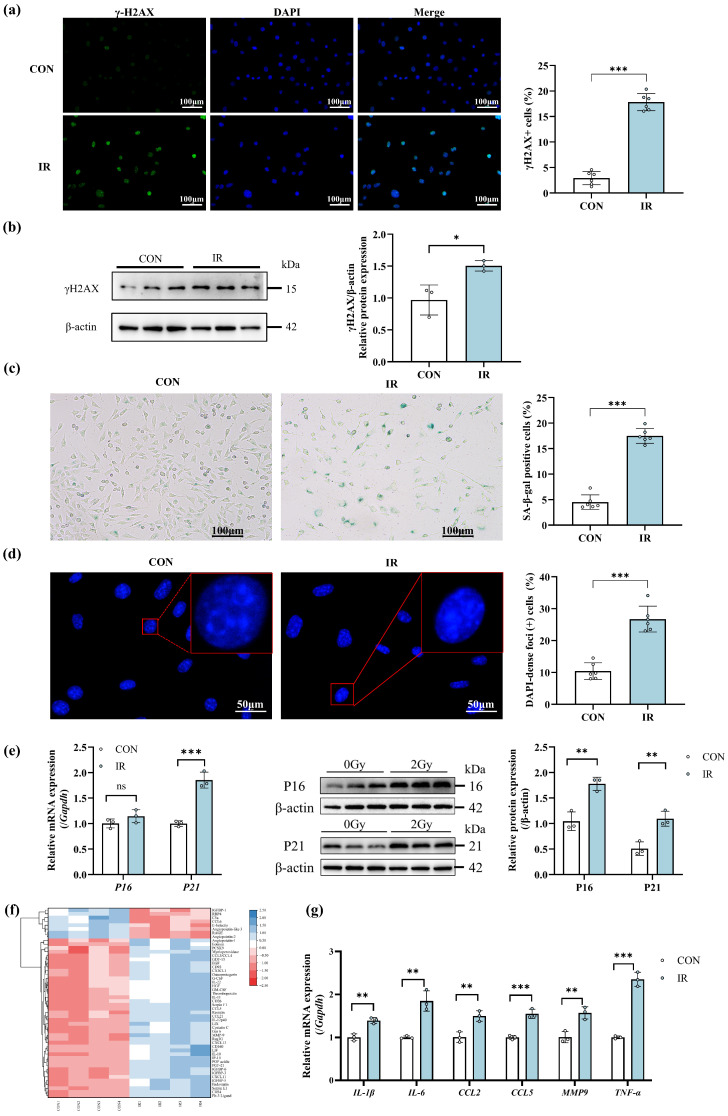
Ionizing radiation triggers persistent DNA damage response and cellular senescence in OCYs. (**a**) Representative γ-H2AX immunofluorescence images of OCY at 24 h after 2 Gy X-ray irradiation (γ-H2AX: green; DAPI: blue; 100× magnification; scale bar = 100 µm) and quantification of the percentage of γ-H2AX-positive nuclei (n = 6); (**b**) relative γ-H2AX protein expression in OCYs at 24 h post-irradiation (normalized to β-actin; n = 3); (**c**) representative SA-β-gal images of OCYs at 72 h post-irradiation (100× magnification, scale bar = 100 µm) and quantification of SA-β-gal-positive cell frequency (n = 6); (**d**) DAPI staining showing condensed punctate DAPI-dense nuclear foci in osteocytes at 72 h post-irradiation (400× magnification, scale bar = 50 µm) and corresponding quantification of cells displaying senescence-associated chromatin condensation-like features (n = 6); (**e**) relative mRNA and protein expression levels of senescence effectors P16^INK4a^ and P21^Cip1/Waf1^ in OCY at 72 h post-irradiation (n = 3); (**f**) hierarchical clustering heatmap of SASP factor secretion profiles in OCY-conditioned medium at 24 h post-irradiation, assessed by cytokine antibody microarray. (**g**) RT-qPCR validation of key SASP factor mRNA expression in OCYs at 24 h post-irradiation. ns: not significant; *p* > 0.05; * *p* < 0.05; ** *p* < 0.01; *** *p* < 0.001.

Accumulation of γ-H2AX is a well-established marker of DNA damage response (DDR) activation [36]. To assess persistent DDR following irradiation, γ-H2AX focus formation was quantified by immunofluorescence microscopy at 24 h post-irradiation, while total protein levels were measured by Western blot. Immunofluorescence analysis revealed a statistically significant increase in γ-H2AX nuclear foci in irradiated osteocytes compared with sham-irradiated controls, indicating sustained DNA damage response signaling and unresolved DSB repair at 24 h after irradiation (Figure 3a). Quantification showed that the proportion of γ-H2AX-positive cells increased 5.08-fold after 2 Gy irradiation (*p* < 0.001, Figure 3a). Consistent with this, Western blot analysis confirmed a 55.1% upregulation of γ-H2AX protein expression (*p* < 0.05, Figure 3b), further supporting persistent activation of the DNA damage response following 2 Gy X-ray exposure.

Persistent DNA damage response signaling is widely recognized as an important contributor to the establishment of cellular senescence, which is a stable cell cycle arrest accompanied by profound transcriptional, metabolic, and structural remodeling. Senescence was therefore evaluated using multiple orthogonal markers: (i) SA-β-gal activity, a widely accepted functional readout reflecting lysosomal biogenesis; (ii) DAPI staining to assess senescence-associated chromatin condensation-like changes; and (iii) expression of canonical senescence effectors (e.g., P16^INK4a^, P21^Cip1/Waf1^). SA-β-gal staining revealed a marked increase in senescent osteocytes following irradiation: the fraction of SA-β-gal-positive cells rose 2.87-fold relative to controls (*p* < 0.001, Figure 3c). Similarly, DAPI staining demonstrated a 2.56-fold elevation in the proportion of cells displaying condensed punctate DAPI-dense nuclear foci after irradiation (*p* < 0.001, Figure 3d). These structures were morphologically distinct from diffuse chromatin staining and were observed together with other senescence-associated phenotypes. Furthermore, the mRNA and protein expression level of the canonical senescence effectors P16^INK4a^ and P21^Cip1/Waf1^ were examined. RT-qPCR analysis revealed that while *P16* mRNA expression exhibited a modest but non-significant increase in irradiated osteocytes relative to the sham-irradiated controls (*p* > 0.05), *P21* mRNA expression was significantly upregulated (*p* < 0.001). Western blot analysis confirmed corresponding increases at the protein level: both P16 and P21 showed significant elevation following 2 Gy X-ray irradiation (*p* < 0.01, Figure 3e). These coordinated transcriptional and translational upregulations provide mechanistic support for radiation-induced cell cycle arrest and stable senescence establishment in osteocytes.

Senescent cells undergo irreversible cell cycle arrest and exhibit markedly reduced proliferative capacity, yet retain robust metabolic activity and secrete a complex, pro-inflammatory milieu known as senescence-associated secretory phenotype (SASP). To comprehensively characterize radiation-induced SASP remodeling in osteocytes, a high-throughput antibody-based proteomic microarray was employed to quantify the secretion levels of 111 cytokines, chemokines, and growth factors in osteocyte-conditioned medium. The analysis revealed that 2 Gy X-ray irradiation profoundly reprogrammed the OCY secretome, with significant upregulation of pro-inflammatory interleukins and chemokines, concomitant with downregulation of select anti-inflammatory and homeostatic cytokines (Figure 3f). To orthogonally validate the high-throughput screening results, RT-qPCR was performed to quantify the mRNA expression levels of six core SASP factors in irradiated OCYs. Consistent with the microarray data, the mRNA expression levels of inflammatory factors, including *IL-1β*, *IL-6*, *CCL2*, *CCL5*, *MMP9*, and *TNF-α*, were significantly elevated in irradiated OCYs versus sham-irradiated controls (*p* < 0.001 or *p* < 0.01, Figure 3g). Collectively, these quantitatively concordant data demonstrate that 2 Gy X-ray irradiation successfully induces a canonical, pro-inflammatory SASP in osteocytes, thereby validating a physiologically relevant in vitro model of radiation-accelerated cellular senescence.

### 3.3. Ionizing Radiation Triggers Sustained Calcium Overload in Osteocytes

**Figure 4:**
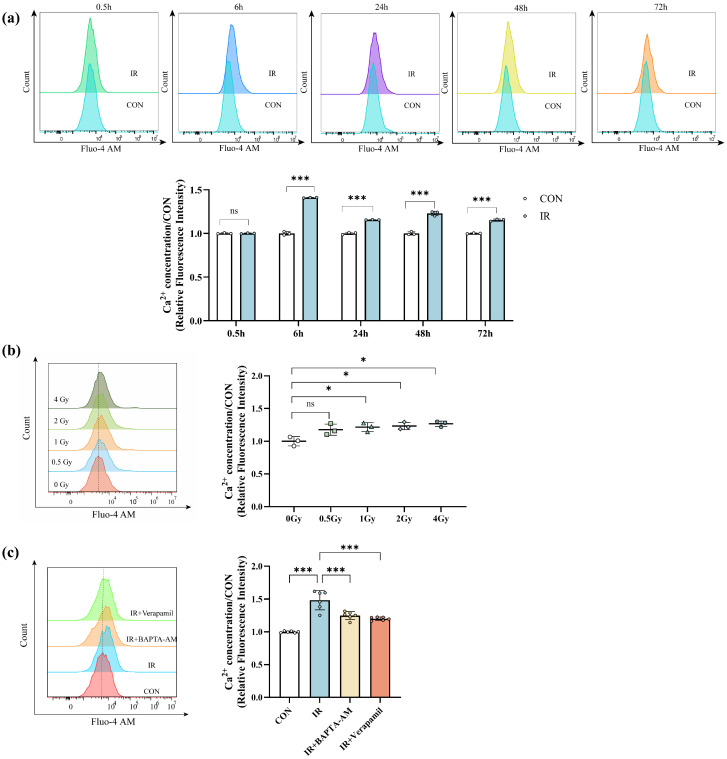
Ionizing radiation induces cytoplasmic calcium overload in osteocytes. (**a**) Time-course analysis of intracellular Ca^2+^ levels in OCYs, quantified by mean fluorescence intensity of Fluo-4 AM staining at 0.5, 6, 24, 48, and 72 h following 2 Gy X-ray irradiation (n = 3); (**b**) dose–response analysis of intracellular Ca^2+^ elevation in OCYs at 6 h post-irradiation with 0.5, 1, 2, or 4 Gy X-rays, assessed via Fluo-4 AM-based flow cytometry (n = 3); (**c**) effects of BAPTA-AM or verapamil treatment on irradiation-induced intracellular Ca^2+^ elevation in osteocytes at 6 h post-irradiation, assessed by Fluo-4 AM fluorescence intensity following 2 Gy irradiation (n = 6). ns: not significant, *p* > 0.05; * *p* < 0.05; *** *p* < 0.001.

To elucidate radiation-induced disruption of calcium homeostasis in osteocytes, the cytoplasmic calcium indicator Fluo-4 AM fluorescent probe was employed to quantify intracellular Ca^2+^ levels following X-ray irradiation across a physiologically relevant dose range of 0.5–4 Gy and a time course of 0.5–72 h. Fluorescence analysis revealed that 2 Gy irradiation elicited a rapid and significant elevation in mean Fluo-4 AM intensity, peaking at 6 h post-exposure (*p* < 0.001, Figure 4a), indicating that ionizing radiation directly induces acute cytosolic calcium overload in osteocytes. Critically, this elevation was not transient; the fluorescence intensity remained significantly elevated at 72 h post-irradiation (*p* < 0.001, Figure 4a), suggesting sustained dysregulation of intracellular Ca^2+^ homeostasis by ionizing radiation. Additionally, a dose–response analysis further showed that cytosolic Ca^2+^ accumulation increased progressively with radiation dose, from 0.5 Gy to 4 Gy (*p* < 0.05, Figure 4b), with maximal elevation observed at 4 Gy, demonstrating that radiation-induced calcium overload exhibits dose dependency. Preliminary dose-finding experiments showed that BAPTA-AM at 10 μM and verapamil at 10 μM did not affect cell viability (Supplementary Figure S1). Therefore, these concentrations were used in subsequent experiments. To further determine whether calcium-modulating interventions could attenuate irradiation-induced cytosolic Ca^2+^ overload, osteocytes were treated with either BAPTA-AM or verapamil prior to irradiation. Both treatments significantly reduced Fluo-4 AM fluorescence intensity in irradiated osteocytes compared with the irradiation-only group (Figure 4c), indicating effective suppression of radiation-induced intracellular Ca^2+^ accumulation. These findings further support the involvement of pathological Ca^2+^ dysregulation in the osteocyte response to ionizing radiation. Collectively, these data suggest that ionizing radiation induces not only premature senescence but also persistent, dose-responsive cytosolic calcium overload in osteocytes.

### 3.4. Pharmacological Inhibition of Calcium Overload Attenuates IR-Induced Osteocyte Senescence

**Figure 5:**
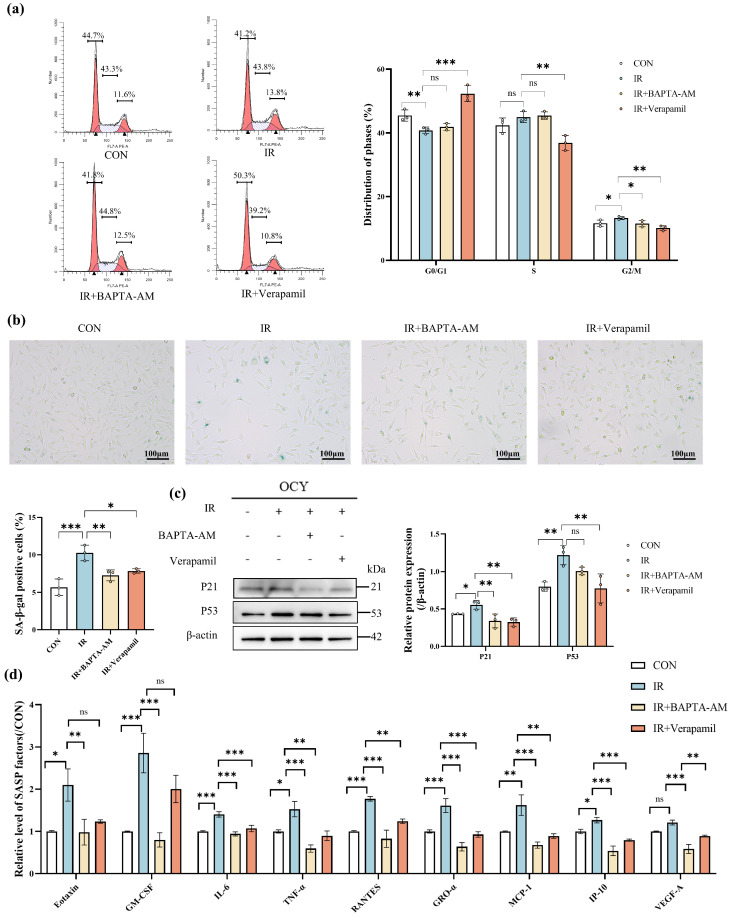
Pharmacological intervention with BAPTA-AM and verapamil alleviates osteocyte senescence. (**a**) Representative flow cytometric profiles and quantitative analysis of cell cycle distribution in osteocytes following BAPTA-AM and verapamil treatments (n = 3); (**b**) representative images (100× magnification, scale bar = 100 µm) and quantitative analysis of SA-β-gal staining positivity in osteocytes following BAPTA-AM and verapamil treatment (n = 3); (**c**) protein expression levels of senescence-associated molecules P21 and P53 in osteocytes following BAPTA-AM and verapamil treatment (n = 3); (**d**) effects of BAPTA-AM and verapamil on SASP factors in conditioned medium from irradiated OCYs. ns: not significant, *p* > 0.05; * *p* < 0.05; ** *p* < 0.01; *** *p* < 0.001.

To determine whether radiation-triggered cytosolic calcium overload is functionally required for IR-induced osteocyte senescence and cell cycle arrest, the membrane-permeant Ca^2+^ chelator BAPTA-AM or the L-type voltage-gated calcium-channel blocker verapamil was employed to pretreat osteocytes prior to 2 Gy X-ray irradiation. Cell cycle distribution was subsequently assessed. The results demonstrated that IR alone induced a significant G2/M phase arrest in osteocytes, increasing the proportion of G2/M cells from 11.6 ± 0.8% in sham-irradiated controls to 13.3 ± 0.4% (*p* < 0.05, Figure 5a). Critically, both BAPTA-AM and verapamil pretreatment fully reversed this effect: BAPTA-AM reduced G2/M levels to 11.5 ± 0.8% (*p* < 0.05 vs. IR group), while verapamil reduced them to 10.2 ± 0.6% (*p* < 0.01 vs. IR group, Figure 5a). Consistent with these findings, SA-β-gal staining revealed that IR increased the fraction of senescent osteocytes by 1.8-fold relative to the controls (*p* < 0.001, Figure 5b). Pharmacological inhibition significantly attenuated this response: BAPTA-AM and verapamil pretreatment significantly reduced SA-β-gal positivity by 28.9% (*p* < 0.01) and 23.4% (*p* < 0.05, Figure 5b), respectively, compared to the irradiated-only group. These complementary functional assays demonstrate that chelating intracellular Ca^2+^ or blocking its influx through L-type channels mitigates key hallmarks of premature senescence—including irreversible cell cycle arrest and lysosomal hyperactivation—thereby establishing a causal role for calcium dysregulation in radiation-accelerated osteocyte senescence.

To further validate the functional protection conferred by BAPTA-AM and verapamil against IR-induced osteocyte senescence, Western blot analysis was performed to quantify the protein expression levels of the canonical senescence regulators P21^Cip1/Waf1^ and P53 in OCY under different treatment conditions. Consistent with prior observations, IR exposure significantly upregulated both P21 (*p* < 0.05) and P53 (*p* < 0.01) in osteocytes relative to the CON group. Notably, BAPTA-AM pretreatment fully reversed P21 upregulation (*p* < 0.01) and attenuated P53 expression. Verapamil pretreatment similarly normalized P21 and P53 protein levels (*p* < 0.01, Figure 5c), indicating potent suppression of the p53–p21 axis. Collectively, these orthogonal pharmacological interventions, targeting either global intracellular Ca^2+^ buffering or L-type channel-mediated Ca^2+^ influx, convergently suppress the core p53–p21 signaling cascade, thereby mechanistically linking calcium dysregulation to the establishment of irreversible cell cycle arrest in irradiated osteocytes.

### 3.5. Pharmacological Inhibition of Calcium Overload Attenuates SASP Secretion in Senescent Osteocytes

Senescent cells develop a pro-inflammatory senescence-associated secretory phenotype (SASP), which can profoundly influence neighboring cells and tissue homeostasis. To assess SASP factor secretion by osteocytes under different experimental conditions, conditioned media were analyzed using cytokine antibody microarrays. IR-treated osteocytes exhibited markedly altered secretion profiles of multiple key pro-inflammatory SASP factors, including Eotaxin, GM-CSF, IL-6, TNF-α, RANTES, GRO-α, MCP-1, IP-10, and VEGF-A, relative to sham-irradiated controls (Figure 5d). IR exposure significantly upregulated all nine factors, with GM-CSF, Eotaxin, and RANTES exhibiting the most pronounced increases: 1.85-fold, 1.09-fold, and 0.77-fold, respectively (*p* < 0.001, Figure 5d).

Critically, pharmacological inhibition of calcium overload, via intracellular Ca^2+^ chelation treatment with BAPTA-AM or L-type calcium-channel blockade with verapamil, significantly suppressed IR-induced expression of these SASP factors (*p* < 0.01–*p* < 0.001; Figure 5d). Notably, both interventions achieved comparable attenuation, supporting a central role for calcium dysregulation not merely as a trigger of senescence but as a direct mediator of the pro-inflammatory secretome. These results collectively demonstrate that calcium overload is a mechanistically upstream driver of SASP acquisition in irradiated osteocytes, thereby implicating Ca^2+^ signaling as a potential therapeutic node to mitigate SASP-mediated disruption of the bone microenvironment.

### 3.6. Pharmacological Inhibition of Calcium Overload Restores Osteoblast–Osteoclast Coupling via Paracrine Signaling

Senescent osteocytes secrete SASP factors that act through autocrine and paracrine mechanisms to dysregulate cellular physiology, thereby sustaining chronic low-grade inflammation and establishing a senescence-associated microenvironment. Within this microenvironment, SASP mediators orchestrate a pathological imbalance between bone resorption and formation by concurrently impairing osteoblast differentiation and promoting osteoclast activation through interconnected signaling pathways. Given the central role of osteocytes as master regulators of bone remodeling, we next investigated whether pharmacological inhibition of IR-induced calcium overload in senescent osteocytes could rescue this dysregulated coupling. To specifically assess the paracrine influence of irradiated osteocytes on bone cell fate decisions, conditioned media were collected from osteocytes subjected to sham irradiation, IR alone, or IR combined with BAPTA-AM or verapamil treatment, and then applied separately to BMSCs (to evaluate osteogenic differentiation) and BMMs (to assess osteoclastogenesis) in distinct co-culture systems, thereby establishing functionally segregated in vitro models of bone formation and resorption.

**Figure 6:**
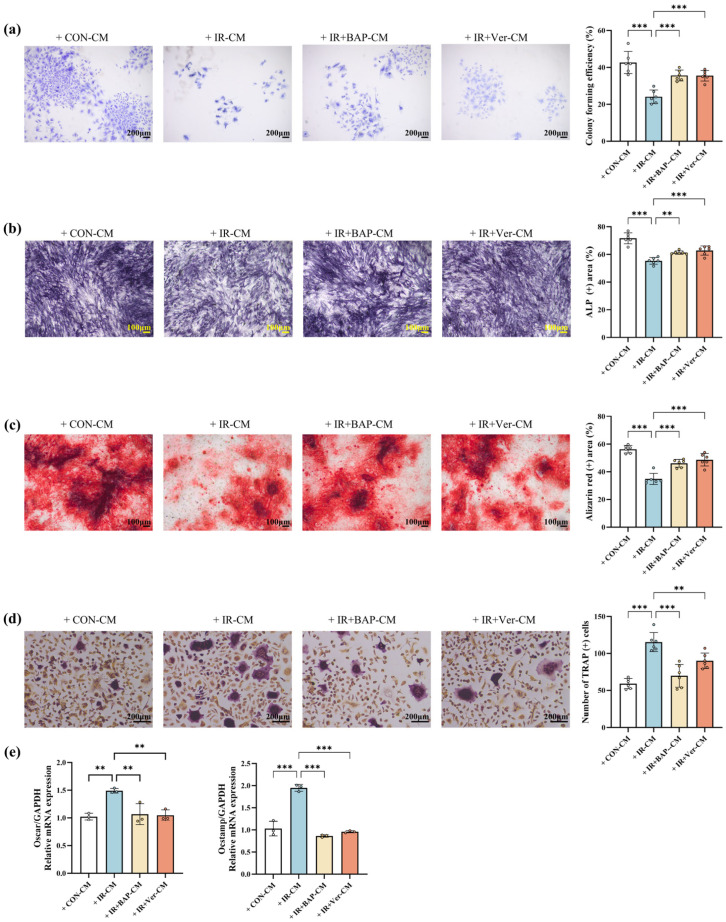
Pharmacological intervention of BAPTA-AM and verapamil rescues balanced osteolineage differentiation in the IR-induced senescent bone microenvironment. (**a**) Representative crystal violet-stained images of BMSC colony-forming units (CFUs) (20× magnification, scale bar = 200 µm), with quantification shown as CFU number per dish (n = 6); (**b**) alkaline phosphatase (ALP) staining of BMSCs under osteogenic induction (100× magnification, scale bar = 100 µm); and quantification reflects integrated ALP-positive areas per dish (n = 6); (**c**) Alizarin Red S staining for extracellular matrix mineralization (100× magnification, scale bar = 100 µm); and quantification represents Alizarin Red-positive areas per dish (n = 6); (**d**) TRAP staining of bone marrow-derived macrophages (BMMs) co-cultured with conditioned medium from irradiated or drug-pretreated osteocytes (40× magnification, scale bar = 100 µm); and quantification represents TRAP-positive, multinucleated osteoclasts (≥3 nuclei per cell) per well (n = 6); (**e**) RT-qPCR analysis of mRNA expression levels of osteoclast-specific genes *Oscar* and *OC-stamp* in BMMs; data normalized to *Gapdh* and expressed relative to the CON-CM group (n = 3). *p* > 0.05; ** *p* < 0.01; *** *p* < 0.001.

The colony-forming unit (CFU) assay was employed to quantitatively assess the self-renewal capacity of BMSCs. As shown in Figure 6a, both qualitative colony formation and quantitative enumeration revealed a marked reduction in the CFU number in the IR-CM group relative to the CON-CM group (*p* < 0.001), demonstrating that conditioned medium from irradiated osteocytes profoundly impairs BMSCs’ clonogenicity. Critically, this impairment was rescued by calcium modulation: colony formation in the IR+BAP-CM and IR+Ver-CM groups increased by 47.7% and 47.4%, respectively (*p* < 0.001, Figure 6a), compared with the IR-CM group, with no statistically significant difference between the two rescue groups, confirming the functional equivalence of intracellular Ca^2+^ chelation and L-type channel blockade.

For osteoblast differentiation, ALP and Alizarin Red S staining showed significantly diminished staining intensity and area in the IR-CM group versus the CON-CM group (*p* < 0.001), indicating robust suppression of both early osteogenic commitment and late-stage mineralization by SASP factors within the senescence-associated microenvironment (Figure 6b,c). Importantly, both BAPTA-AM and verapamil pretreatment of irradiated osteocytes restored these deficits: the IR+BAP-CM group exhibited 10.8% (*p* < 0.01) and 32.3% (*p* < 001) increases in ALP-positive and Alizarin Red-positive areas, while the IR+Ver-CM group showed increases of 13.4% and 39.6% (both *p* < 0.001, Figure 6b,c). Collectively, these data establish that calcium overload is a mechanistically upstream determinant of SASP-mediated suppression of BMSC osteogenic potential and that its targeted inhibition fully restores clonogenicity, differentiation initiation, and matrix mineralization capacity.

TRAP staining revealed that bone marrow-derived macrophages (BMMs) co-cultured with conditioned medium from irradiation-induced senescent osteocytes (IR-CM) formed significantly more TRAP-positive, multinucleated osteoclasts than those co-cultured with conditioned medium from non-irradiated control osteocytes (CON-CM). A quantitative analysis demonstrated 115.4 ± 11.7 TRAP-positive osteoclasts per well in the IR-CM group, approximately 1.95-fold higher than the CON-CM group (*p* < 0.001, Figure 6d), providing direct morphological evidence of hyperactivated osteoclastogenesis in the irradiation-induced senescent bone microenvironment. Critically, this pathological enhancement was reversed by modulating calcium signaling: conditioned medium from irradiated osteocytes pretreated with the intracellular Ca^2+^ chelator BAPTA-AM (IR+BAP-CM) reduced TRAP-positive osteoclast formation by 39.6% relative to the IR-CM group (*p* < 0.001), restoring levels to near those of the CON-CM group. Similarly, conditioned medium from irradiated osteocytes pretreated with the L-type voltage-gated calcium-channel blocker verapamil (IR+Ver-CM) induced a significant 21.9% reduction (90.08 ± 9.59 TRAP-positive cells per well, *p* < 0.01, Figure 6d). A complementary RT-qPCR analysis confirmed that IR-CM upregulated transcription of the osteoclast-specific genes *OC-stamp* and *Oscar* in BMMs, key molecular markers of osteoclast commitment and maturation. Importantly, both BAPTA-AM and verapamil pretreatments abolished this upregulation, suppressing *OC-stamp* and *Oscar* expression to baseline levels (*p* < 0.01 and *p* < 0.001, Figure 6e). Together, these data demonstrate that calcium overload is a central mechanistic driver of dysregulated osteoclastogenesis in the bone niche. By rescuing both quantitative osteoclast formation and qualitative gene expression profiles, calcium modulation restores functional homeostasis, thereby linking the attenuation of osteocyte senescence and SASP secretion to the preservation of balanced osteoblast–osteoclast lineage allocation in irradiated bone.

### 3.7. Calcium Overload Activates the NFATc1-ATF3 Signaling Pathway in Irradiated Osteocytes

**Figure 7:**
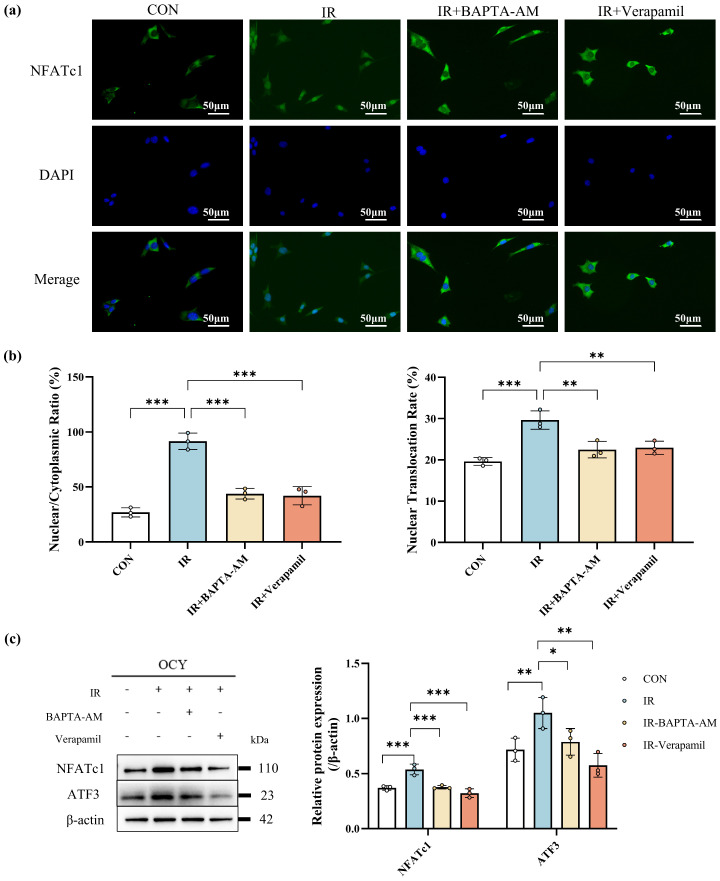
Calcium overload mediates activation of the NFATc1/ATF3 axis in irradiated osteocytes. (**a**) Representative immunofluorescence images showing NFATc1 (green) subcellular localization in osteocytes, nuclei were stained with DAPI (blue) (200× magnification, scale bar = 50 µm); (**b**) quantification of NFATc1 nuclear-to-cytoplasmic fluorescence intensity ratio (left panel) and percentage of osteocytes exhibiting nuclear NFATc1 accumulation (right panel) (n = 3); (**c**) Western blot analysis of protein expression levels of NFATc1 and ATF3 protein expression in osteocytes (n = 3). *p* > 0.05; * *p* < 0.05; ** *p* < 0.01; *** *p* < 0.001.

To elucidate whether calcium overload mechanistically couples irradiation to osteocyte senescence, the activation status of the NFATc1-ATF3 signaling axis, a canonical calcium-responsive pathway implicated in cellular stress adaptation and senescence regulation, was interrogated. Immunofluorescence analysis revealed that in non-irradiated osteocytes (OCYs), NFATc1 was predominantly cytoplasmic, with minimal nuclear accumulation. In contrast, exposure to 2 Gy X-ray irradiation induced robust nuclear translocation of NFATc1: the nuclear/cytoplasmic fluorescence intensity ratio dramatically increased relative to the CON group (*p* < 0.001, Figure 7a). Quantitative assessment further confirmed a significant rise in the proportion of NFATc1-positive nuclei, from 19.62 ± 0.75% in controls to 29.65 ± 1.82% in irradiated cells (*p* < 0.01, Figure 7b), providing direct evidence that ionizing radiation triggered functional activation of NFATc1 via calcium-dependent nuclear import.

Critically, this irradiation-induced NFATc1 activation was abolished by calcium signal modulation. Both BAPTA-AM and verapamil pretreatment fully prevented NFATc1 nuclear accumulation: the nuclear/cytoplasmic ratio in the IR+BAPTA-AM and IR+Verapamil groups was reduced to levels indistinguishable from the CON group (*p* < 0.001 vs. IR group, Figure 7a,b), and nuclear translocation rates dropped to 20.14 ± 1.27% and 22.89 ± 1.53%, respectively.

Collectively, these data verify that ionizing radiation drives the nuclear activation of NFATc1 via intracellular calcium overload. Interruption of calcium signaling by either BAPTA-AM or verapamil effectively suppresses IR-induced aberrant nuclear translocation of NFATc1.

Concomitant with NFATc1 nuclear translocation, Western blot analysis revealed robust upregulation of both NFATc1 and ATF3 protein expression in irradiated osteocytes (*p* < 0.001 and *p* < 0.01) relative to non-irradiated controls. This coordinated induction, occurring downstream of calcium-dependent nuclear import, supports a functional cascade in which ionizing radiation-induced calcium overload triggers not only NFATc1 activation but also its transcriptional output, including ATF3, a well-established mediator of oxidative stress response and senescence execution. Critically, pretreatment with either BAPTA-AM or verapamil significantly attenuated the IR-induced upregulation of NFATc1 and ATF3 protein expression (*p* < 0.05–*p* < 0.001, Figure 7c), confirming that these protective effects are specifically mediated by disruption of intracellular calcium signaling. Collectively, these data establish calcium overload as necessary and sufficient for activation of the NFATc1-ATF3 axis in irradiated osteocytes. Calcium modulation restores NFATc1 localization and ATF3 expression downstream, disrupting the full pathological cascade rather than isolated components alone. The Ca^2+^/NFATc1/ATF3 signaling axis thus emerges as a concrete therapeutic target in radiation-induced bone senescence, with clear mechanistic support.

## 4. Discussion

Radiation-induced bone injury poses a substantial clinical challenge, as ionizing radiation (IR)-induced bone loss predisposes patients to fragility fractures, impaired fracture healing, and elevated risk of skeletal metastasis [3,5,6]. Osteocytes, the most abundant and longest-lived mechanosensory cells embedded within the mineralized bone matrix, are now established as central orchestrators of skeletal homeostasis [37]. However, their responses to IR remain underexplored relative to osteoblasts and osteoclasts. In clinical radiotherapy, fractionated regimens commonly deliver single doses of 1.8–2.5 Gy, which can induce sublethal damage in adjacent normal tissues, provoking sustained oxidative stress and low-grade chronic inflammation, key drivers of persistent cellular senescence [38]. To model this clinically relevant exposure, primary murine osteocytes were subjected to a single 2 Gy X-ray dose, thereby establishing a physiologically grounded IR-induced osteocyte senescence model. Moreover, 2 Gy provided a sublethal irradiation condition suitable for studying senescence-associated alterations in surviving osteocytes, as Annexin V/PI analysis showed only a modest increase in apoptosis after irradiation. Nevertheless, this in vitro model does not fully recapitulate the biological effects of higher single-dose irradiation or repeated fractionated irradiation regimens, which may induce distinct cellular responses and should be further examined in future studies. Functional assays revealed that IR robustly suppressed osteocyte proliferation, induced G2/M-phase cell cycle arrest, and elicited dendritic atrophy characterized by significant shortening. Osteocytes reside in lacunae and extend slender, gap junction-coupled dendritic processes through canaliculi to form a vast, three-dimensional syncytium. This network serves three interdependent functions: (i) sensing mechanical strain via fluid shear stress; (ii) enabling metabolic cooperation and intercellular ion exchange; and (iii) secreting paracrine regulators, including sclerostin (SOST) and receptor activator of NF-κB ligand (RANKL), which coordinately govern bone formation and resorption [37]. IR-induced dendritic shortening directly compromises the structural integrity of this network, disrupting both physical connectivity among osteocytes and their functional crosstalk with osteoblasts, osteoclasts, and the extracellular matrix. Consequently, impaired mechanotransduction and dysregulated paracrine signaling emerge as early pathogenic events that initiate and propagate microenvironmental imbalance. Clinically, the delayed onset of osteoporosis, nonunion fractures, and osteonecrosis in irradiated patients reflects this dual collapse of osteocyte-mediated mechanosensation and endocrine regulation.

As the longest-lived cells in bone tissue, osteocytes lack regenerative capacity, rendering them vulnerable to the accumulation of unrepaired damage and predisposing them to persistent functional decline and irreversible senescence. Cellular senescence is a heterogeneous and multifactorial state. Therefore, its robust identification requires validation across multiple molecular and phenotypic hallmarks, rather than dependence on any single biomarker [39]. To rigorously characterize the IR-induced osteocyte senescence model, we adopted a multiparametric assessment strategy based on concordant evidence from multiple complementary senescence-associated indicators, including persistent DNA damage response (γ-H2AX foci), increased lysosomal activity assessed by SA-β-gal staining, upregulation of cell cycle regulators such as P16^INK4a^ and P21^Cip1/Waf1^, SASP factor secretion and supportive morphological evidence of chromatin condensation-like changes reflected by condensed punctate DAPI-dense nuclear foci. This approach avoids overreliance on any single assay and better reflects the heterogeneous, multifactorial nature of cellular senescence. Consistent with this strategy, 2 Gy X-ray irradiation significantly increased γ-H2AX foci, SA-β-gal activity, the proportion of cells displaying condensed punctate DAPI-dense nuclear foci, and the transcription and translation of P16^INK4a^ and P21^Cip1/Waf1^. Importantly, the DAPI-dense nuclear foci were interpreted only as supportive morphological observations of senescence-associated chromatin condensation-like changes, rather than definitive evidence of genuine SAHF formation. Because γ-H2AX foci were assessed at 24 h after irradiation, these data should not be interpreted as reflecting the initial burden of acute DNA damage. Instead, γ-H2AX positivity at this time point more likely represents persistent DNA damage response signaling associated with delayed or incomplete repair. Collectively, these findings support the establishment of a senescence-associated phenotype in primary osteocytes following exposure to a single 2 Gy dose of ionizing radiation [40]. Notably, although irradiation also induced a measurable increase in apoptotic cells, the overall cellular response under the present experimental conditions was predominantly characterized by senescence-associated alterations rather than extensive apoptotic cell death. Annexin V/PI flow cytometric analysis demonstrated that apoptosis increased from approximately 8.3% in control cells to 11.1% in cells subjected to 2 Gy irradiation, suggesting that the selected irradiation intensity primarily induced persistent but sublethal cellular stress. Such stress conditions are more consistent with sustained DNA damage responses, prolonged cell cycle arrest, and the establishment of stress-induced premature senescence rather than overwhelming apoptotic elimination. Mechanistically, IR-induced DNA double-strand breaks may activate the ATM/ATR–Chk1/Chk2 kinase cascade, leading to stabilization and activation of P53, which transcriptionally induces P21. P21 can inhibit the Cyclin B1–CDK1 complex and contribute to G2/M arrest, thereby preventing mitotic progression in cells with damaged DNA. While initially protective, prolonged G2/M arrest in post-mitotic osteocytes may transition into pathological senescence. Critically, sustained p16^INK4a^ expression drives irreversible cell cycle exit by inhibiting CDK4/6 mediated phosphorylation of RB, thereby blocking E2F transcription factors in an inactive, hypophosphorylated state, thereby establishing stable cell cycle arrest [41]. The concurrent upregulation of both p16^INK4a^ and p21^Cip1/Waf1^ observed herein indicates synergistic engagement of the p53-p21 and p16-RB tumor suppressor axes, an integrative mechanism previously documented in radiation-induced senescence of fibroblasts and hematopoietic stem cells, and now extended to the osteocyte lineage.

Although senescent cells undergo irreversible cell cycle arrest, they remain metabolically active and secrete SASP, a complex, evolutionarily conserved signaling program comprising pro-inflammatory cytokines, chemokines, matrix metalloproteinases, bioactive lipids, growth factors, non-coding nucleotides, and extracellular vesicles [39,42]. SASP operates through autocrine and paracrine mechanisms to establish a self-reinforcing inflammatory niche; it sustains local chronic inflammation, induces DNA damage and telomere dysfunction in neighboring cells via reactive oxygen species (ROS) and NF-κB activation, and impairs tissue repair, thereby actively propagating aging and degeneration beyond the initially damaged cells [43]. In this study, cytokine antibody microarray profiling of irradiated osteocyte-conditioned medium revealed a robust induction of SASP, characterized by significant upregulation of canonical pro-inflammatory mediators, including IL-6, IL-1β, CCL2, CCL5, MMP9 and TNF-α. Within the bone microenvironment, these SASP factors directly stimulate osteoclastogenesis of osteoclast precursors while concurrently suppressing osteoblast maturation, establishing a self-amplifying pathological loop. Thus, irradiated osteocytes function not merely as passive victims of radiation but as active instigators of microenvironmental collapse, positioning osteocyte senescence as a pivotal mechanistic bridge between acute ionizing radiation exposure and the delayed onset of degenerative bone pathologies.

Beyond canonical senescence markers, this study uncovered a previously underappreciated early perturbation: ionizing radiation (IR) induces rapid and sustained cytosolic Ca^2+^ overload in osteocytes, a dysregulation that precedes hallmark senescence phenotypes and implicates calcium signaling as a critical upstream driver of IR-induced osteocyte senescence. Tight spatiotemporal control of intracellular Ca^2+^ homeostasis is indispensable for mitochondrial bioenergetics, redox balance, and cell fate decisions. In addition to plasma membrane calcium influx, intracellular Ca^2+^ homeostasis is also tightly regulated by endoplasmic reticulum (ER)-associated calcium buffering proteins such as calreticulin. Although the present study did not specifically investigate ER calcium regulatory mechanisms, the potential involvement of calreticulin in radiation-induced osteocyte Ca^2+^ dysregulation and senescence warrants further investigation. Under basal conditions, osteocytes maintain low cytosolic Ca^2+^ levels, whereas physiological mechanical stimuli trigger transient, oscillatory Ca^2+^ signals via Piezo1 activation or PLC-IP3-mediated ER Ca^2+^ release, thereby fine-tuning the expression of bone anabolic genes and catabolic regulators [44]. This study revealed that 1–4 Gy IR provoked significant cytosolic Ca^2+^ elevation within 6 h, before the onset of typical senescence phenotypes. This temporal precedence positions Ca^2+^ overload not as a consequence but as a mechanistic initiator of the senescence cascade. Even 0.5 Gy elicited measurable Ca^2+^ dysregulation, suggesting that calcium signaling may serve as a sensitive indicator for early radiation-induced osteocyte dysfunction. Importantly, pharmacological intervention experiments further strengthened this conclusion. Both the intracellular Ca^2+^ chelator BAPTA-AM and the L-type calcium-channel blocker verapamil significantly attenuated Fluo-4 AM fluorescence intensity in irradiated osteocytes, demonstrating that these interventions effectively attenuated IR-induced cytosolic Ca^2+^ accumulation.

The functional contribution of Ca^2+^ dysregulation to osteocyte senescence was further supported by the observation that normalization of intracellular Ca^2+^ homeostasis markedly attenuated irradiation-associated senescence phenotypes. Both treatments markedly attenuated IR-induced senescence, including reversing G2/M phase arrest, reducing the frequency of SA-β-gal-positivity cells, and downregulating P21 and P53 protein expression. Critically, the efficacy of verapamil demonstrates that pathological Ca^2+^ entry via L-type VGCCs contributes substantially to IR-triggered cytosolic Ca^2+^ dysregulation in osteocytes. While BAPTA-AM has been shown to mitigate apoptosis in HepG-2 cells by restoring global Ca^2+^ homeostasis, and verapamil is known to protect against Ca^2+^ overload-induced cytotoxicity in cardiomyocytes, their convergent suppression of senescence phenotypes in irradiated osteocytes underscores the specificity and centrality of Ca^2+^ overload, not secondary toxicity, as the primary driver of this pathological cascade. The concordant rescue effects observed with these two functionally distinct agents provide compelling, mechanism-based evidence that cytosolic Ca^2+^ overload is a necessary and early pathogenic node in radiation-induced osteocyte senescence.

The impact of calcium modulation on SASP secretion was further investigated. IR exposure robustly upregulated the secretion of key SASP factors, including IL-6, MCP-1 (CCL2), and RANTES (CCL5), in osteocyte-conditioned medium. Critically, this radiation-induced SASP amplification was markedly attenuated by both BAPTA-AM and verapamil. These SASP factors orchestrate bone microenvironmental dysregulation through non-redundant, synergistic mechanisms: (i) IL-6, TNF-α, and GM-CSF directly enhance osteoclastogenesis by stimulating RANKL expression in stromal/osteoblastic cells and promoting survival and differentiation of osteoclast precursors; (ii) chemokines including CCL5 (RANTES), CCL2 (MCP-1), CXCL10 (IP-10), and CXCL1 (GRO-α), recruit monocytes/macrophages to irradiated bone, amplifying local inflammation and osteoclast activation; (iii) MMP-3 degrades collagen and non-collagenous matrix proteins, undermining structural integrity and releasing matrix-bound growth factors that further exacerbate resorption; and (iv) VEGF-A not only promotes angiogenesis but also directly enhances osteoclast precursor survival and fusion via VEGFR1 signaling under inflammatory conditions. This study demonstrates that IR-induced osteocyte senescence is intrinsically coupled to this multifaceted SASP program and that pharmacological normalization of cytosolic Ca^2+^ overload disrupts this coupling at its origin, leading to coordinated downregulation of all major SASP classes. Consequently, calcium-targeted intervention has a dual action mechanism: it mitigates cell-autonomous senescence in osteocytes and interrupts paracrine-driven microenvironment collapse, thereby representing a mechanistically grounded strategy to prevent radiation-induced bone degeneration.

Osteocytes function as the central regulators of bone remodeling homeostasis, precisely coordinating osteoblast and osteoclast activity through the secretion of multiple soluble signaling molecules. Among these, sclerostin (SOST), a protein highly and specifically expressed by osteocytes, acts as a potent negative regulator of the Wnt/β-catenin pathway, thereby suppressing osteoblast differentiation and bone matrix deposition [18]. Concurrently, osteocytes are a primary cellular source of both RANKL and its decoy receptor OPG; dynamic shifts in the RANKL/OPG ratio directly govern the rate of osteoclastogenesis [18]. To model the senescence-associated microenvironment induced by IR-mediated osteocyte senescence and to determine whether targeting calcium overload can indirectly restore bone homeostasis by modulating SASP, conditioned media (CM) from osteocytes across experimental groups were collected and applied to co-culture systems with BMSCs (osteoblast precursors) and BMMs (osteoclast precursors). In the osteogenic co-culture system, BMSCs exposed to CM from irradiated osteocytes exhibited significantly impaired self-renewal capacity and reduced osteogenic differentiation potential, as demonstrated by diminished ALP activity and defective mineralized nodule formation. Notably, pretreatment of irradiated osteocytes with the intracellular calcium chelator BAPTA-AM or the L-type calcium-channel blocker verapamil partially rescued these deficits: BMSCs co-cultured with CM from pretreated osteocytes showed restored self-renewal and multilineage differentiation capacity. These results indicate that calcium overload-targeted intervention alleviates the inhibitory effects of the IR-induced senescent microenvironment on osteogenesis by normalizing the SASP profile secreted by senescent osteocytes. In parallel, in the osteoclastic co-culture system, BMMs treated with CM from irradiated osteocytes displayed markedly enhanced osteoclastogenic differentiation, evidenced by increased numbers of TRAP-positive multinucleated cells and upregulated expression of key osteoclast markers (Oscar and OC-stamp). This pro-osteoclastogenic effect was significantly attenuated when BMMs were exposed to CM from irradiated osteocytes pretreated with BAPTA-AM or verapamil, demonstrating that calcium modulation suppresses the secretion of pro-osteoclastogenic SASP factors from senescent osteocytes. Conditioned medium-based co-culture systems are well-established tools for dissecting paracrine communication. In this study, CM from IR-induced senescent osteocytes exerted a dual pathological effect, suppressing osteoblastogenesis while promoting osteoclastogenesis, indicating that the SASP contains both osteogenesis-inhibitory cytokines (e.g., IL-6, TNF-α) and osteoclastogenesis-stimulatory factors (e.g., RANKL, M-CSF). These findings suggest that osteocyte senescence functions as a mechanistic bridge connecting intracellular calcium dysregulation to bone microenvironment deterioration following irradiation. Targeting calcium overload appears to rebalance bone homeostasis by downregulating this pathogenic SASP signature, a conclusion strongly supported by our quantitative measurements of SASP factor secretion.

NFATc1, a calcium-responsive transcription factor and member of the NFAT family (comprising NFATc1-c4), was originally identified as a master regulator of T-cell activation and differentiation. In quiescent cells, NFATc1 is sequestered in the cytoplasm in a hyperphosphorylated, transcriptionally inactive state. Upon elevation of intracellular Ca^2+^, Ca^2+^ binds calmodulin, triggering calcineurin phosphatase activation. Calcineurin then dephosphorylates NFATc1, exposing its nuclear localization signal and enabling rapid nuclear translocation to drive transcription of target genes. The immunofluorescence analysis in this study revealed that ionizing radiation (IR) induces both nuclear accumulation and upregulated expression of NFATc1 in osteocytes, providing direct evidence of IR-mediated NFATc1 activation. Beyond immune regulation, NFATc1 has been identified as a key mediator of cellular senescence. In H_2_O_2_-induced senescent hepatocytes, NFATc1 expression is significantly upregulated and correlates temporally with increased levels of established senescence markers, including p16^INK4a^, NF-κB activity, and core SASP factors (IL-1β, IL-6, IL-8). Mechanistically, NFATc1 amplifies SASP production and secretion through the NF-κB/TMP21 axis [45]. Consistent with these observations, the IR-induced alterations in SASP secretion reported here further corroborate NFATc1 activation and its functional role in SASP dysregulation in irradiated osteocytes. Importantly, NFATc1 serves as a central signaling node that links perturbed calcium homeostasis to cell senescence. Both Ca^2+^ release from endoplasmic reticulum and Ca^2+^ influx across the plasma membrane converge on activation of the calcineurin/NFAT pathway, which in turn stimulates p53-dependent and p53-independent senescence programs. Furthermore, NFATc1 directly binds the promoter of ITPR2, which encodes the inositol 1,4,5-trisphosphate receptor type 2, a major ER Ca^2+^ release channel, thereby establishing a self-reinforcing feedback loop that sustains aberrant calcium signaling and perpetuates the senescence state [46]. In this study, pharmacological inhibition of calcium signaling with either BAPTA-AM or verapamil effectively prevented IR-induced NFATc1 nuclear translocation in osteocytes. Collectively, these findings indicate that NFATc1 acts as a calcium-sensitive signaling mediator linking radiation-induced Ca^2+^ dysregulation to downstream transcriptional responses associated with osteocyte senescence.

Among the downstream transcriptional targets potentially regulated by NFATc1, ATF3 emerged as a particularly relevant candidate in the present study. ATF3 is a stress-inducible, AP-1-associated transcription factor whose roles in regulating cell proliferation, differentiation, and stress adaptation are well established [47]. Importantly, ATF3 has been increasingly recognized as a stress-responsive transcription factor involved in the regulation of cellular senescence. In human umbilical vein endothelial cells (HUVECs), exogenous ATF3 binds directly to open chromatin regions near senescence-associated loci, including CD44, where it recruits histone acetyltransferases to deposit H3K27ac marks, thereby promoting chromatin relaxation, transcriptional activation, and the establishment of senescence programs; this leads to robust induction of p16^INK4a^ and elevated SA-β-gal activity [48]. Furthermore, ATF3 binds and transactivates specific senescence-associated endogenous retroviral elements such as LTR39 and MER61C, triggering dsRNA accumulation, thereby activating antiviral innate immune signaling and contributing to pro-inflammatory SASP production [49]. Collectively, these findings establish ATF3 as a multifaceted epigenetic and transcriptional orchestrator of senescence. In our study, IR significantly upregulates ATF3 protein expression. Notably, pharmacological inhibition of calcium overload using either BAPTA-AM or verapamil significantly attenuated IR-induced ATF3 upregulation in osteocytes. This identifies dysregulated calcium signaling as an upstream regulator of ATF3 expression and provides mechanistic evidence linking radiation-induced Ca^2+^ dyshomeostasis to transcriptional reprogramming in osteocytes [50,51]. Given that inhibition of calcium signaling simultaneously suppressed NFATc1 activation and ATF3 expression, our findings suggest that ATF3 may function downstream of calcium-dependent NFATc1 signaling in irradiated osteocytes. Critically, the identification of the NFATc1-ATF3 signaling axis as an essential downstream pathway reveals a novel, mechanism-based point for intervention. Calcineurin inhibitors, such as the clinically approved immunosuppressants tacrolimus (FK506) and cyclosporin A, directly suppress NFATc1 activation and can be tested in rigorous preclinical evaluation for their capacity to prevent IR-induced osteocyte senescence and associated bone loss [52].

The clinical safety profile of the L-type calcium-channel blocker verapamil is well established through decades of use in cardiovascular medicine, and its therapeutic potential for mitigating bone injury, particularly radiation-induced bone loss, is supported by emerging preclinical evidence. In ovariectomized (OVX) mice, daily intraperitoneal injection of verapamil at 2.11–4.22 mg/kg for six weeks significantly preserved trabecular bone microarchitecture, with the mechanism involving downregulation of thioredoxin-interacting protein (Txnip) and reduced oxidative stress [53]. Given its established dosing regimens, pharmacokinetics and tolerability in humans, verapamil may represent a promising candidate for repurposing to the treatment of radiation-induced osteocyte dysfunction and bone injury. While BAPTA-AM remains a preclinical tool due to its limited stability, its potent ability to buffer intracellular Ca^2+^ provides a compelling proof of concept for calcium-targeted strategies. Advanced nano-delivery platforms, such as ROS-responsive hyaluronic acid-bilirubin nanoparticles and dextran-phenylboronic acid-silymarin nanocarriers, have been developed to enable spatiotemporally controlled drug release in ROS-enriched injured tissues [54]. These systems may provide a conceptual framework for localized delivery of calcium-modulating agents to radiation-damaged bone. Importantly, the calcium-targeted interventions investigated in the present study were intended to attenuate pathological intracellular Ca^2+^ overload rather than to interfere with physiological calcium required for bone mineralization. Nevertheless, the long-term skeletal safety of systemic calcium-modulating strategies warrants careful evaluation in future in vivo and translational studies. Localized or bone-targeted delivery approaches may help minimize potential off-target effects while preserving physiological calcium homeostasis.

Several limitations should be acknowledged. First, although dose-gradient CCK-8 assays indicated that 10 μM BAPTA-AM and 10 μM verapamil did not cause overt cytotoxicity or impair basal metabolic activity in non-irradiated osteocytes, these experiments did not assess potential drug-specific effects on senescence markers, SASP secretion, or NFATc1/ATF3 signaling. Therefore, the absence of full drug-only control groups for these downstream endpoints remains a limitation of the present study, and inclusion of such controls in future investigations would help confirm that the observed protective effects are attributable to modulation of radiation-induced Ca^2+^ dysregulation rather than drug-independent effects. Secondly, although the present data confirm that L-type calcium channels are involved in the IR-induced calcium influxes in osteocytes, the precise molecular mechanisms underlying radiation-triggered channel activation remain incompletely defined. Future studies should clarify the distinct contributions of individual L-type channel subtypes (e.g., Cav1.2, Cav1.3) to IR-induced osteocyte senescence, thereby enabling the rational design of subtype-selective pharmacological interventions. Finally, while in vitro co-culture systems provided initial evidence for the paracrine influence of senescent osteocytes, these simplified systems do not fully capture the multicellular complexity and dynamic crosstalk within the bone microenvironment. Beyond osteocytes, the bone niche contains vascular endothelial cells, immune cells, and bone marrow adipocytes, all of which respond to IR by secreting regulatory factors or releasing extracellular vesicles that modulate bone turnover and inflammatory responses [22]. Consequently, rigorous in vivo validation in physiologically relevant animal models will be essential to corroborate these mechanistic findings, assess the functional impact of calcium overload-targeted interventions on systemic bone homeostasis, and establish a robust preclinical foundation for translating these strategies into clinical applications for radiation-induced bone senescence.

## 5. Conclusions

This study provides the first mechanistic evidence that ionizing radiation (IR) induces osteocyte senescence via calcium overload-dependent activation of the NFATc1-ATF3 signaling axis. A single 2 Gy X-ray irradiation provoked sustained intracellular Ca^2+^ elevation in osteocytes, initiating a cascade wherein calcineurin dephosphorylates NFATc1, enabling its nuclear translocation and transcriptional activation of ATF3, ultimately driving canonical senescence hallmarks, including persistent cell cycle arrest (p16^INK4a^/p21^Cip1/Waf1^ upregulation), elevated SA-β-gal activity, and robust SASP secretion. Critically, pharmacological targeting of calcium overload through intracellular Ca^2+^ chelation with BAPTA-AM or selective L-type calcium-channel blockade with verapamil not only abrogates NFATc1 nuclear accumulation and ATF3 induction but also rescues osteocyte viability, restores physiological paracrine control over bone remodeling, and re-establishes balanced osteoblast–osteoclast coupling. These findings support the concept that pathological calcium overload is an important upstream pathogenic trigger of IR-induced osteocyte dysfunction, and suggest that the NFATc1-ATF3 axis may represent a therapeutically relevant signaling pathway. Consequently, interventions that normalize calcium homeostasis or disrupt this axis represent a promising strategy for mitigating radiation-induced bone loss in patients undergoing radiotherapy, thereby preserving skeletal health and enhancing long-term functional outcomes, although further in vivo validation and safety evaluation are required.

## Figures and Tables

**Table 1:** Primer sequence for RT-qPCR.

Gene Name	Forward Primer (5′-3′)	Reverse Primer (5′-3′)
*PCNA*	GAACCTCACCAGCATGTCCA	ATTCACCCGACGGCATCTTT
*E11*	GTTTTGGGGAGCGTTTGGTTC	CATTAAGCCCTCCAGTAGCAC
*P16*	CGCAGGTTCTTGGTCACTGT	TGTTCACGAAAGCCAGAGCG
*P21*	CCTGGTGATGTCCGACCTG	CCATGAGCGCATCGCAATC
*CCL2*	GTCTGTGCTGACCCCAAGAAG	TGGTTCCGATCCAGGTTTTTA
*CCL5*	GCCCACGTCAAGGAGTATTTCT	ACAAACACGACTGCAAGATTGG
*IL-1β*	GATCGTCGCTATAACCCTCCAA	TGGGATCTGTGTGGCATAAGAG
*IL-6*	CCCTGGGAAGCTGTTATCTTCA	CTGATGGGCTTCAGCACAGA
*MMP9*	CCGAAGTCATAGCCACACTCAA	CAAGGGAGCTTCAGGGTCAAG
*TNF-α*	TCAGAATGAGGCTGGATAAG	GGAGGCAACAAGGTAGAG
*OC-stamp*	AACTCAGCTCTGCCATGAAGT	ACACTGCTCCAGGAAGATGAT
*Oscar*	GCACCCCTGCACATAGTCAGA	AGGACAGTGCTCCTGATGGAA
*G* *apdh*	AGGTCGGTGTGAACGGATTTG	GGGGTCGTTGATGGCAACA

**Table 2:** Antibodies for Western blotting.

Antibody Against	Cat.No	Dilution	Provider	Location
E11	ab11936	1:1000	Abcam	Cambridge, MA, USA
γ-H2AX	ab81299	1:1000	Abcam	Cambridge, MA, USA
P16^INK4a^	ab51243	1:1000	Abcam	Cambridge, MA, USA
P21^Cip1/Waf1^	ab109199	1:1000	Abcam	Cambridge, MA, USA
P53	ab26	1:1000	Abcam	Cambridge, MA, USA
NFATc1	ab25916	1:1000	Abcam	Cambridge, MA, USA
ATF3	ab207434	1:1000	Abcam	Cambridge, MA, USA
β-actin	4970	1:1000	Cell Signaling Technology Inc.	Danvers, MA, USA

## Data Availability

The original contributions presented in this study are included in the article/Supplementary Material. Further inquiries can be directed to the corresponding author.

## Supplementary Materials

The following supporting information can be downloaded at: https://www.mdpi.com/article/10.3390/life16060984/s1, Figure S1. Effects of BAPTA-AM and verapamil alone on basal osteocyte viability/metabolic activity.

## Abbreviations

The following abbreviations are used in this manuscript:

IR	Ionizing radiation
SASP	Senescent cell-associated secretory phenotype
SIPS	Stress-induced premature senescence
SA-β-gal	Senescence-associated-β-galactosidase
SNCs	Senescent cells
DSBs	DNA double-strand breaks
DDR	DNA damage response
ER	Endoplasmic reticulum
CaMKII	Ca^2+^/Calmodulin-Dependent Protein Kinase II
NFATc1	Nuclear factor of activated T-cells, cytoplasmic 1
ATF3	Activating transcription factor 3
CDKIs	Cyclin-dependent kinase inhibitors
BMSCs	Bone marrow-derived mesenchymal stem cells
BMMS	Bone marrow-derived macrophages
EdU	5-ethynyl-2′-deoxyuridine
PFA	Polyoxymethylene
ALP	Alkaline Phosphatase
TRITC	Tetramethylrhodamine isothiocyanate
M-CSF	Macrophage colony-stimulating factor
RANKL	Receptor activator of NF-kB ligand
CFU	Colony-forming unit
TRAP	Tartrate-resistant acid phosphatase
Oscar	Osteoclast-associated receptor
OC-stamp	Osteoclast stimulatory transmembrane protein
BSA	Bovine serum albumin
SOST	Sclerostin
ROS	Reactive oxygen species
VGCC	Voltage-gated calcium channel
PCNA	Proliferating cell nuclear antigen
HUVECs	Human umbilical vein endothelial cells

## References

[1] McBride W.H., Schaue D. (2020). Radiation-Induced Tissue Damage and Response. J. Pathol..

[2] Curi M.M., Cardoso C.L., de Lima H.G., Kowalski L.P., Martins M.D. (2016). Histopathologic and Histomorphometric Analysis of Irradiation Injury in Bone and the Surrounding Soft Tissues of the Jaws. J. Oral Maxillofac. Surg..

[3] Hoveidaei A., Karimi M., Khalafi V., Fazeli P., Hoveidaei A.H. (2024). Impacts of Radiation Therapy on Quality of Life and Pain Relief in Patients with Bone Metastases. World J. Orthop..

[4] Quillen E.E., Schaaf G.W., Justice J.N., Dugan G.O., Johnson B., Reed C., Olson J.D., Cline J.M. (2025). Widespread Multimorbidity in a Cohort of Aging, Radiation-Exposed Rhesus Macaques. Radiat. Res..

[5] Sauer K., Zizak I., Forien J.-B., Rack A., Scoppola E., Zaslansky P. (2022). Primary Radiation Damage in Bone Evolves via Collagen Destruction by Photoelectrons and Secondary Emission Self-Absorption. Nat. Commun..

[6] Guo W., Hoque J., Garcia Garcia C.J., Spiller K.V., Leinroth A.P., Puviindran V., Potnis C.K., Gunn K.A., Newman H., Ishikawa K. (2023). Radiation-Induced Bone Loss in Mice Is Ameliorated by Inhibition of HIF-2α in Skeletal Progenitor Cells. Sci. Transl. Med..

[7] Lončar Brzak B., Horvat Aleksijević L., Vindiš E., Kordić I., Granić M., Vidović Juras D., Andabak Rogulj A. (2023). Osteonecrosis of the Jaw. Dent. J..

[8] Jelin-Uhlig S., Weigel M., Ott B., Imirzalioglu C., Howaldt H.-P., Böttger S., Hain T. (2024). Bisphosphonate-Related Osteonecrosis of the Jaw and Oral Microbiome: Clinical Risk Factors, Pathophysiology and Treatment Options. Int. J. Mol. Sci..

[9] Pignolo R.J., Law S.F., Chandra A. (2021). Bone Aging, Cellular Senescence, and Osteoporosis. JBMR Plus.

[10] Li K., Hu S., Chen H. (2025). Cellular Senescence and Other Age-Related Mechanisms in Skeletal Diseases. Bone Res..

[11] Nelson G., Wordsworth J., Wang C., Jurk D., Lawless C., Martin-Ruiz C., von Zglinicki T. (2012). A Senescent Cell Bystander Effect: Senescence-Induced Senescence. Aging Cell.

[12] Farr J.N., Xu M., Weivoda M.M., Monroe D.G., Fraser D.G., Onken J.L., Negley B.A., Sfeir J.G., Ogrodnik M.B., Hachfeld C.M. (2017). Targeting Cellular Senescence Prevents Age-Related Bone Loss in Mice. Nat. Med..

[13] Li X., Chen M., Zhang Y., Li J., Xiang L., Xiao Y., Xiang Y., Chen L., Ran Q., Li Z. (2026). A Review of Ionizing Radiation-Induced Senescence of Bone Marrow Mesenchymal Stem/Stromal Cells: Mechanisms and Therapeutic Strategies. Curr. Issues Mol. Biol..

[14] Ibragimova M., Kussainova A., Aripova A., Bersimbaev R., Bulgakova O. (2024). The Molecular Mechanisms in Senescent Cells Induced by Natural Aging and Ionizing Radiation. Cells.

[15] Russo M., Spagnuolo C., Moccia S., Tedesco I., Lauria F., Russo G.L. (2021). Biochemical and Cellular Characterization of New Radio-Resistant Cell Lines Reveals a Role of Natural Flavonoids to Bypass Senescence. Int. J. Mol. Sci..

[16] Hu W., Liang J.-W., Liao S., Zhao Z.-D., Wang Y.-X., Mao X.-F., Hao S.-W., Wang Y.-F., Zhu H., Guo B. (2021). Melatonin Attenuates Radiation-Induced Cortical Bone-Derived Stem Cells Injury and Enhances Bone Repair in Postradiation Femoral Defect Model. Mil. Med. Res..

[17] Richardson K.K., Ling W., Krager K., Fu Q., Byrum S.D., Pathak R., Aykin-Burns N., Kim H.-N. (2022). Ionizing Radiation Activates Mitochondrial Function in Osteoclasts and Causes Bone Loss in Young Adult Male Mice. Int. J. Mol. Sci..

[18] Wu Y., Gan D., Liu Z., Qiu D., Tan G., Xu Z., Xue H. (2025). Osteocytes: Master Orchestrators of Skeletal Homeostasis, Remodeling, and Osteoporosis Pathogenesis. Front. Cell Dev. Biol..

[19] Zhao F., Han H., Wang J., Wang J., Zhai J., Zhu G. (2025). Oversecretion of CCL3 by Irradiation-Induced Senescent Osteocytes Mediates Bone Homeostasis Imbalance. Cells.

[20] Guan J., Li T., Ma F., Wang N., Zhang H., Li J., Li J., Xu C., Liu Q. (2025). DNA Damage-Dependent Mechanisms of Ionizing Radiation-Induced Cellular Senescence. PeerJ.

[21] Milani M., Pihan P., Hetz C. (2023). Calcium Signaling in Lysosome-Dependent Cell Death. Cell Calcium.

[22] Terrell K., Choi S., Choi S. (2023). Calcium’s Role and Signaling in Aging Muscle, Cellular Senescence, and Mineral Interactions. Int. J. Mol. Sci..

[23] Qin L., Liu W., Cao H., Xiao G. (2020). Molecular Mechanosensors in Osteocytes. Bone Res..

[24] Sun A., Yang J., Chang Y., Yang S. (2025). NFATc1 Activates the Ras/Raf/P38 MAPK Pathway to Promote the Progression of Lung Adenocarcinoma. Transl. Cancer Res..

[25] Pan H.-Y., Ladd A.V., Biswal M.R., Valapala M. (2021). Role of Nuclear Factor of Activated T Cells (NFAT) Pathway in Regulating Autophagy and Inflammation in Retinal Pigment Epithelial Cells. Int. J. Mol. Sci..

[26] Wang S., Qian H., Zhang L., Liu P., Zhuang D., Zhang Q., Bai F., Wang Z., Yan Y., Guo J. (2021). Inhibition of Calcineurin/NFAT Signaling Blocks Oncogenic H-Ras Induced Autophagy in Primary Human Keratinocytes. Front. Cell Dev. Biol..

[27] Katz H.R., Arcese A.A., Bloom O., Morgan J.R. (2022). Activating Transcription Factor 3 (ATF3) Is a Highly Conserved Pro-Regenerative Transcription Factor in the Vertebrate Nervous System. Front. Cell. Dev. Biol..

[28] Bagheri Z., Abuei H., Jaafari A., Taki S., Arsanjani A.A., Farhadi A. (2025). Inhibition of P16 and NF-κB Oncogenic Activity in Human Papillomavirus-Infected Cervical Cancer Cells: A New Role for Activating Transcription Factor-3. Yale J. Biol. Med..

[29] Liu S., Li Z., Lan S., Hao H., Baz A.A., Yan X., Gao P., Chen S., Chu Y. (2024). The Dual Roles of Activating Transcription Factor 3 (ATF3) in Inflammation, Apoptosis, Ferroptosis, and Pathogen Infection Responses. Int. J. Mol. Sci..

[30] Rohini M., Haritha Menon A., Selvamurugan N. (2018). Role of Activating Transcription Factor 3 and Its Interacting Proteins under Physiological and Pathological Conditions. Int. J. Biol. Macromol..

[31] Li H., Zhang F., Zhang C., Zhou M., Liu Q., Zeng G. (2025). Silencing ATF3 Mediates Mitochondrial Homeostasis and Improves Ischemic Stroke through Regulating the MAPK Signaling Pathway. Front. Mol. Neurosci..

[32] Bae W.-J., Lee S.-I. (2025). Activating Transcription Factor 3 (ATF3) Regulates Cellular Senescence and Osteoclastogenesis via STAT3/ERK and P65/AP-1 Pathways in Human Periodontal Ligament Cells. Int. J. Mol. Sci..

[33] Kim K.-H., Park B., Rhee D.-K., Pyo S. (2015). Acrylamide Induces Senescence in Macrophages through a Process Involving ATF3, ROS, P38/JNK, and a Telomerase-Independent Pathway. Chem. Res. Toxicol..

[34] Rohini M., Vairamani M., Selvamurugan N. (2021). TGF-Β1-Stimulation of NFATC2 and ATF3 Proteins and Their Interaction for Matrix Metalloproteinase 13 Expression in Human Breast Cancer Cells. Int. J. Biol. Macromol..

[35] He F., Bai J., Wang J., Zhai J., Tong L., Zhu G. (2019). Irradiation-Induced Osteocyte Damage Promotes HMGB1-Mediated Osteoclastogenesis in Vitro. J. Cell Physiol..

[36] Raavi V., Perumal V., Paul S.F.D. (2021). Potential Application of γ-H2AX as a Biodosimetry Tool for Radiation Triage. Mutat. Res. Rev. Mutat. Res..

[37] Yuze M., Hu J., Jun L., Cheng X., Tianwen X., Junqiang Z. (2025). Osteocytes Function as Biomechanical Signaling Hubs Bridging Mechanical Stress Sensing and Systemic Adaptation. Front. Physiol..

[38] Rodier F., Campisi J. (2011). Four Faces of Cellular Senescence. J. Cell Biol..

[39] Gorgoulis V., Adams P.D., Alimonti A., Bennett D.C., Bischof O., Bishop C., Campisi J., Collado M., Evangelou K., Ferbeyre G. (2019). Cellular Senescence: Defining a Path Forward. Cell.

[40] Tilton M., Liao J., Kim C., Shaygani H., Potes M.A., Cordova D.J., Kirkland J.L., Miller K.M. (2025). Tracing Cellular Senescence in Bone: Time-Dependent Changes in Osteocyte Cytoskeleton Mechanics and Morphology. Small.

[41] Rubin S.M., Sage J., Skotheim J.M. (2020). Integrating Old and New Paradigms of G1/S Control. Mol. Cell.

[42] Zhang L., Pitcher L.E., Yousefzadeh M.J., Niedernhofer L.J., Robbins P.D., Zhu Y. (2022). Cellular Senescence: A Key Therapeutic Target in Aging and Diseases. J. Clin. Investig..

[43] Hofbauer L.C., Lademann F., Rauner M. (2023). Deconstructing Cellular Senescence in Bone and Beyond. J. Clin. Investig..

[44] Matsuura K., Saeki T., Takahashi T., Torigoe T., Watarai K., Osaki A., Hojyo T. (2021). Bilateral Femoral Head Osteonecrosis in a Patient with Metastatic Breast Cancer Receiving Long-term Zoledronic Acid Treatment: A Case Report. Mol. Clin. Oncol..

[45] Chen Z., Huang L., Ding L., Zhang C., Li Y., Wang B., Shi J., Zhang J. (2025). NFATc1 Facilitates Hepatocellular Carcinoma Progression by Regulating the Senescence-Associated Secretory Phenotype. Sci. Rep..

[46] Martin N., Zhu K., Czarnecka-Herok J., Vernier M., Bernard D. (2023). Regulation and Role of Calcium in Cellular Senescence. Cell Calcium.

[47] Jeong B.-C., Kim J.H., Kim K., Kim I., Seong S., Kim N. (2017). ATF3 Modulates Calcium Signaling in Osteoclast Differentiation and Activity by Associating with C-Fos and NFATc1 Proteins. Bone.

[48] Zhang C., Zhang X., Huang L., Guan Y., Huang X., Tian X.-L., Zhang L., Tao W. (2021). ATF3 Drives Senescence by Reconstructing Accessible Chromatin Profiles. Aging Cell.

[49] Mao J., Zhang Q., Zhuang Y., Zhang Y., Li L., Pan J., Xu L., Ding Y., Wang M., Cong Y.-S. (2024). Reactivation of Senescence-Associated Endogenous Retroviruses by ATF3 Drives Interferon Signaling in Aging. Nat. Aging.

[50] Rodier F., Coppé J.-P., Patil C.K., Hoeijmakers W.A.M., Muñoz D.P., Raza S.R., Freund A., Campeau E., Davalos A.R., Campisi J. (2009). Persistent DNA Damage Signalling Triggers Senescence-Associated Inflammatory Cytokine Secretion. Nat. Cell Biol..

[51] Fenech M., Kirsch-Volders M., Natarajan A.T., Surralles J., Crott J.W., Parry J., Norppa H., Eastmond D.A., Tucker J.D., Thomas P. (2011). Molecular Mechanisms of Micronucleus, Nucleoplasmic Bridge and Nuclear Bud Formation in Mammalian and Human Cells. Mutagenesis.

[52] Zeng M., Zhu Z., Yuan W., Tang Z., Qing Z., Lu Q., Wu X., He J., Li Y., Li Z. (2024). Verapamil Inhibits Inflammation and Promotes Autophagy to Alleviate Ureteral Scar by Regulation of CaMK IIδ/STAT3 Axis. Ren. Fail..

[53] Cao X., Rong K., Li Y., Zhang P., Liu K., Cui L., Fu S., Hua Q., Yang X., Zhang H. (2025). A Novel Application Perspective of the Clinical-Used Drug Verapamil on Osteoporosis via Targeting Txnip. J. Orthop. Transl..

[54] Wang Y., Pu M., Yan J., Zhang J., Wei H., Yu L., Yan X., He Z. (2023). 1,2-Bis(2-Aminophenoxy)Ethane-N,N,N′,N′-Tetraacetic Acid Acetoxymethyl Ester Loaded Reactive Oxygen Species Responsive Hyaluronic Acid-Bilirubin Nanoparticles for Acute Kidney Injury Therapy via Alleviating Calcium Overload Mediated Endoplasmic Reticulum Stress. ACS Nano.

