# Supplementary material for: Intracellular Calcium Overload Promotes NFATc1-ATF3 Activation and Induces the Senescence-Associated Phenotype in Irradiated Osteocytes
Source: Life (Basel). 2026 Jun 11;16(6):984. doi: 10.3390/life16060984 (PMC13301319; doi:10.3390/life16060984)
Supplement: Supplementary file 1 [file life-16-00984-s001.zip › Supplementary Figure S1.pdf]

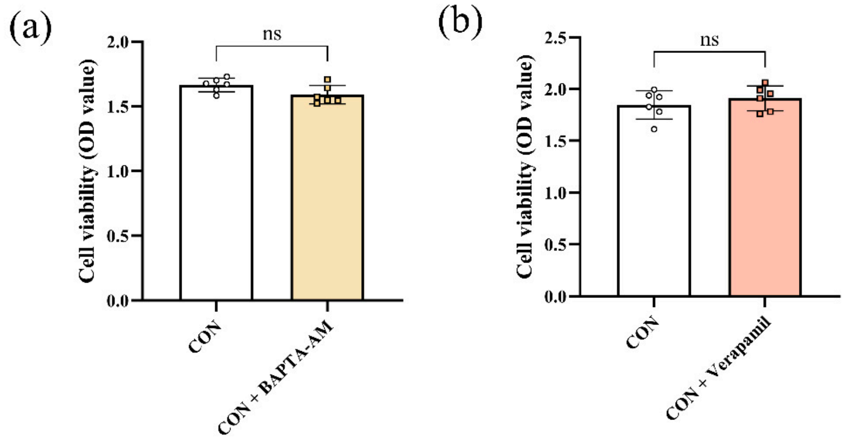

Supplementary Figure S1. Effects of BAPTA-AM and verapamil alone on basal osteocyte viability/metabolic activity. CCK-8 assays were performed in non-irradiated osteocytes treated with BAPTA-AM or verapamil alone at the concentration used in the irradiation-intervention experiments. (a) Cellular viability assessed by CCK-8 assay in untreated control and BAPTA-AM-treated osteocytes. (n = 6); (b) cellular viability assessed by CCK-8 assay in untreated control and verapamil-treated osteocytes. ns: not significant.
